# Supplementary material for: Diversity in striatal synaptic circuits arises from distinct embryonic progenitor pools in the ventral telencephalon
Source: Cell Rep. 2021 Apr 27;35(4):109041. doi: 10.1016/j.celrep.2021.109041 (PMC8097690; doi:10.1016/j.celrep.2021.109041)
Supplement: Document S1. Figures S1–S4 and Tables S1–S4 [file mmc1.pdf]

Cell Reports, Volume *35*

Supplemental information

Diversity in striatal synaptic circuits arises from distinct embryonic progenitor pools in the ventral telencephalon

Fran van Heusden, Anežka Macey-Dare, Jack Gordon, Rohan Krajeski, Andrew Sharott, and Tommas Ellender

Supplemental Figures

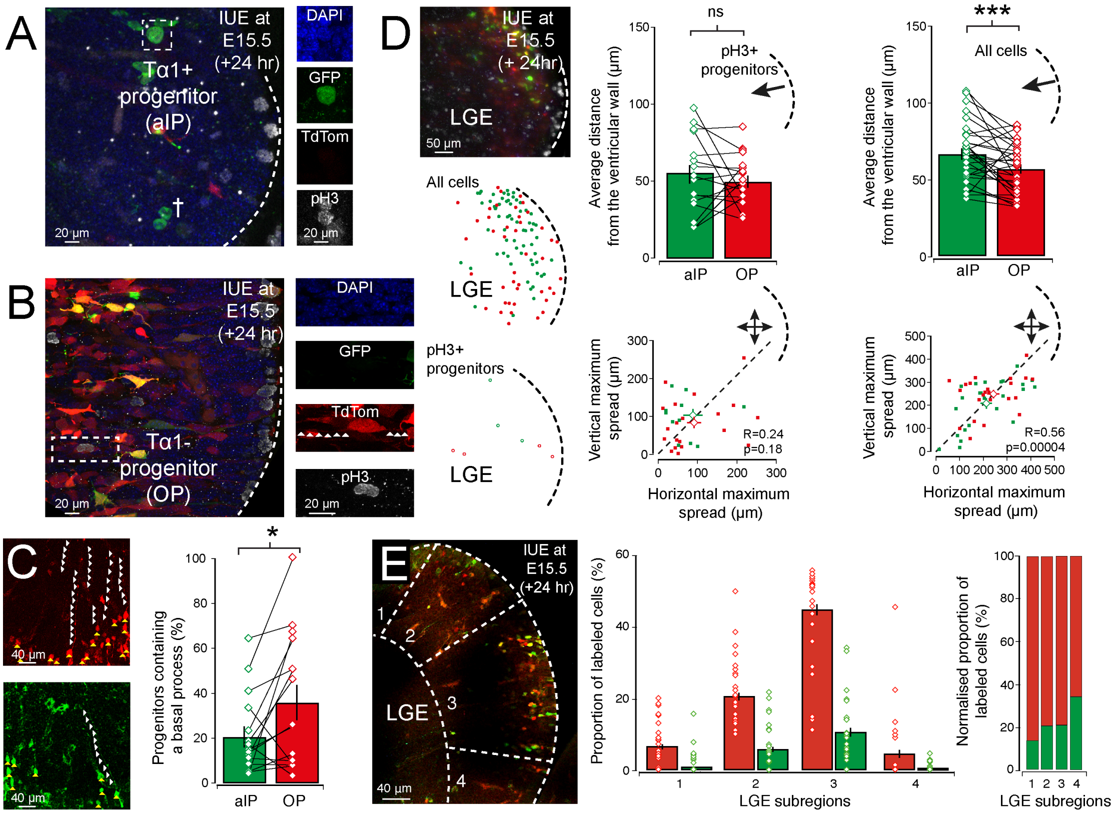

Figure S1: Spatial distribution and morphology of IUE labeled cells in the LGE. Related to Figure 1. (**A**) Actively dividing Tα1$^+$/GFP$^+$ progenitors with a short rounded morphology and lacking a basal process, referred here as apical intermediate progenitors (aIP), could be found at both the ventricular wall or, as shown here, also at a slight distance from the ventricular wall, reminiscent of subapical progenitors (Pilz *et al.*, 2013). Cross symbol indicates Tα1$^+$/GFP$^+$ progenitor undergoing cytokinesis. (**B**) Dividing Tα1$^-$/TdTom$^+$ progenitors, referred here as other progenitors (OP), often retained a basal process during division and could similarly be found at the ventricular wall or, as shown here, at a distance from the ventricular wall, reminiscent of bipolar radial glial cells (Pilz *et al.*, 2013). (**C**) Quantification of progenitors in the LGE proliferative zones exhibiting a basal process. Note that significantly more Tα1$^-$/TdTom$^+$ cells retained a basal process. (**D**) Measurements of the average distance from the ventricular wall and overall spread for Tα1$^+$/GFP$^+$ and Tα1$^-$/TdTom$^+$ cells within the LGE. We find that both Tα1$^+$/GFP$^+$ and Tα1$^-$/TdTom$^+$ cells are intermixed in the LGE (left). These cells consist of both dividing progenitors expressing the mitotic marker phospho-histone H3 (pH3$^+$) and non-dividing progenitors and young neurons (pH3$^-$). Whereas there is no difference in the average distance from the ventricular wall amongst dividing (pH3$^+$) Tα1$^+$/GFP$^+$ and Tα1$^-$/TdTom$^+$ progenitors (middle), the Tα1$^+$/GFP$^+$ non-dividing progenitors and young neurons are located significantly further away from the ventricular wall 24h after IUE (right). (**E**) Example coronal section of embryonic LGE 24 hours after IUE at E15.5 with Tα1-Cre and FLEx plasmids demonstrating that GFP$^+$ and TdTom$^+$ labeled cells can be seen in all progenitor domains of the LGE. Bar plots of the average proportion of Tα1$^+$/GFP$^+$ and Tα1$^-$/TdTom$^+$ labeled cells in the different progenitor domains of the LGE (middle). Note that the labeling strategy predominantly targeted LGE3, but that all progenitor domains contain labeled cells (all labeled cells: LGE1: 7.6%, LGE2: 32.0%, LGE3: 59.0% and LGE4: 1.3%, n = 17 mice). The relative proportion of Tα1$^+$/GFP$^+$ and Tα1$^-$/TdTom$^+$ labeled cells appears relatively constant between the different domains of the LGE (right).

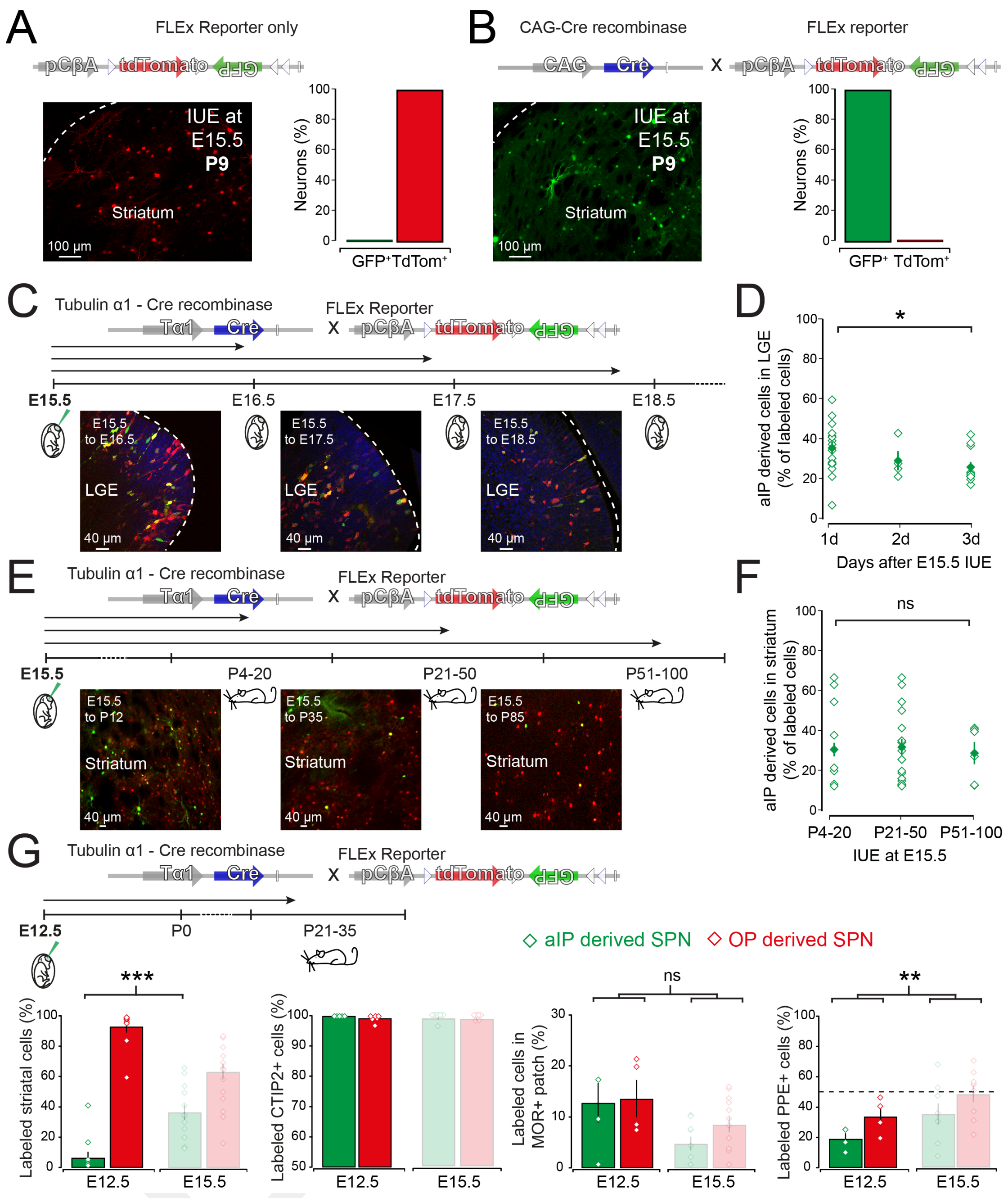

Figure S2: Faithful reporting of embryonic Tα1 expression. Related to Figure 1. (**A**) IUE at E15.5 of the CβA-FLEx reporter plasmid alone resulted in only TdTom^{+} striatal SPNs at postnatal day (P)9 (n = 292/4 mice). This excludes the possibility of spontaneous recombination of the CβA-FLEx reporter. (**B**) IUE at E15.5 of the CβA-FLEx reporter plasmid and a second plasmid, in which a ubiquitous promoter ('CAG-Cre') drove Cre recombinase, resulted in complete and rapid recombination and only GFP^{+} striatal SPNs were observed at P9 (n = 196/4 mice). (**C**) Tα1-Cre and CβA-FLEx plasmids were delivered by IUE at embryonic age E15.5 and GFP^{+} and TdTom^{+} cells were imaged in the LGE 24h, 48h or 72h later. (**D**) GFP^{+} and TdTom^{+} cells were quantified in LGE at 1d, 2d or 3d after IUE at E15.5. In support of the idea that the majority of Cre-mediated recombination of the reporter plasmid occurred within 24 h following IUE, the proportion of GFP^{+} cells in the LGE remained relatively stable during embryonic development with a small but significant decrease in the number of GFP^{+} cells ($p = 0.045$, Kruskal-Wallis test; n = 1204/17, 535/4 and 620/11 at 1d, 2d and 3d after IUE at E15.5). (**E**) GFP^{+} and TdTom^{+} striatal neurons were imaged across a range of postnatal ages after IUE at E15.5. (**F**) The proportion of GFP^{+} aIP-derived striatal neurons did not change significantly across a wide range of postnatal ages ($p = 0.99$, Kruskal-Wallis test, n = 3743/20, 2677/24 and 345/6 mice at

P4-20, P21-50, P51-100) after IUE at E15.5, consistent with the idea that there was no further recombination of the reporter plasmid postnatally. (**G**) IUE at the early embryonic age E12.5 of Tα1-Cre and CβA-FLEx plasmids resulted in significantly lower numbers of GFP^+ aIP-derived striatal neurons in postnatal (P21-35) striatum (E12.5: 6.6 ± 4.0% and E15.5: 31.6 ± 3.4%, Mann-Whitney test, p=0.0002, n = 657/10 and 2677/24 mice) as compared to IUE at E15.5, supporting the idea that Tα1-expressing progenitors form a considerable proliferating population during later periods of neurogenesis. The majority of progenitor derived neurons labeled at this embryonic age expressed the SPN marker CTIP2 (E12.5 aIP: 100.0 ± 0.0% and E12.5 OP: 99.2 ± 0.5%, n = 275/4 mice) and could be found in both (MOR)-rich patches and MOR-poor matrix compartments (E12.5 aIP/MOR^+: 12.8 ± 3.9% and OP/MOR^+: 13.6 ± 3.7%, 370/4 mice, middle) with a trend towards larger numbers of early labeled SPNs in (MOR)-rich patches (E12.5: 12.4 ± 2.4% vs. E15.5: 6.9 ± 1.3% in MOR^+ patches; Mann-Whitney test, p = 0.170, n = 370/4 and 427/12 mice). Grouped together the E12.5 IUE labeled SPNs contained lower numbers of PPE^+/iSPNs as compared to those labeled at E15.5 (E12.5 vs. E15.5, Mann-Whitney test; p = 0.006; n = 275/4 and 254/9 mice, right) consistent with the idea that dSPNs have an earlier birthdate.

DRAFT

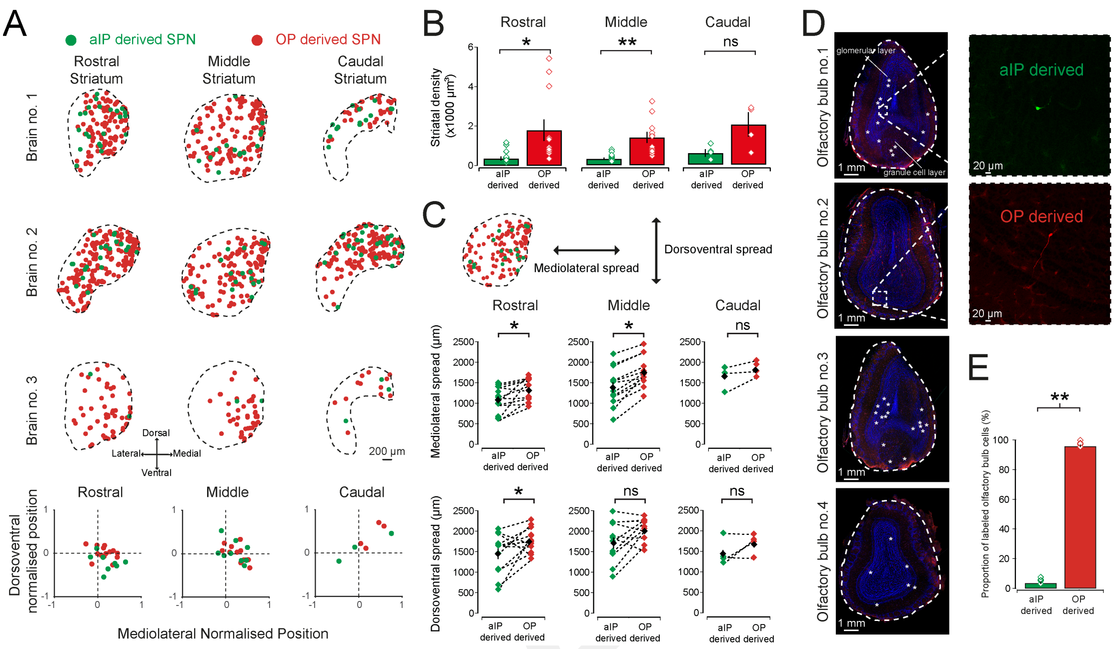

Figure S3: Labeled aIP and OP-derived striatal SPNs in the rostral, central, and caudal striatal regions and olfactory bulb. Related to Figure 1. (**A**) Distribution of aIP and OP-derived SPNs (P21-35) in the rostral, middle, and caudal regions of the striatum in three different IUE brains. Labeled progenitor-derived SPNs were predominantly found in the medial aspects of the striatum. Labeled SPNs were only counted within a 10-µm plane of a brain section (see **Methods**), which for the dataset reported in this figures was on average 13.8 ± 1.9 aIP-derived SPNs and 60.5 ± 9.4 OP-derived SPNs per 10-µm plane of a brain section. (**B**) The average relative densities of aIP and OP-derived SPNs in rostral, middle, and caudal regions of striatum were similar, with an overall higher density of OP-derived SPNs. (**C**) Analysis of the maximum spread of aIP and OP-derived SPNs in the mediolateral and dorsoventral axes shows that OP-derived SPNs exhibit a larger spread than aIP-derived SPNs in both axes. (**D**) IUE of embryonic progenitors in the LGE also labeled a small number of, predominantly OP-derived, olfactory bulb cells. Example images of four different olfactory bulbs from IUE mice with labeled cells indicated by asterisks. High magnification images of an aIP-derived cell (right, top) and OP-derived cell (right, bottom) in the olfactory bulb. (**E**) The majority of labeled cells in the olfactory bulbs were OP-derived (aIP: 3.9 ± 1.6% and OP: 96.1 ± 1.6%, $p=0.0079$, Mann-Whitney test, $n= 191/ 5$ olfactory bulbs).

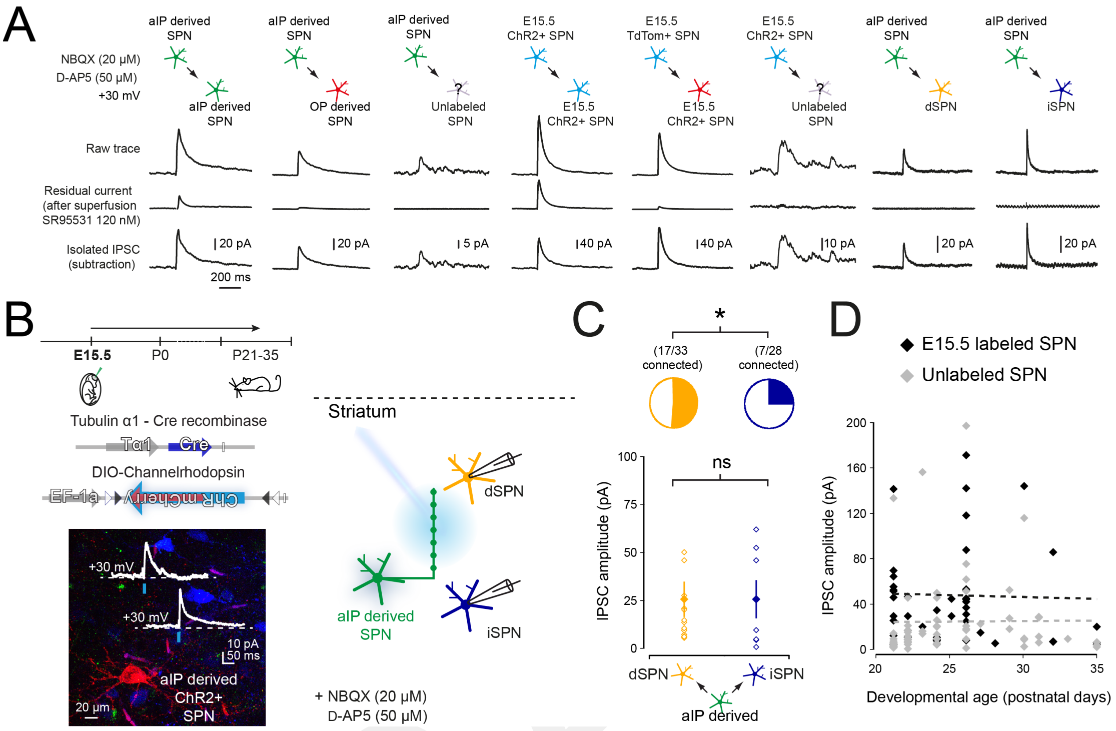

Figure S4: Optogenetic study of local inhibitory connections between SPNs. Related to Figure 4. (**A**) Example responses recorded from striatal SPNs after photo-activation of ChR2-expressing presynaptic SPNs. All recordings were made at a holding potential of +30 mV in the presence of blockers of excitatory transmission. Plotted are the raw responses to photo-activation (top), the residual responses to photo-activation after additional superfusion of low concentrations of the $GABA_A$-receptor antagonist SR95531 (200nM) (middle), and the subtracted responses which corresponded to the $GABA_A$ receptor-mediated response or IPSC used for comparisons of inhibitory synaptic strength (bottom). (**B**) IUE of Tα1-Cre and DIO-ChR2-mCherry plasmids allowed for the expression of ChR2-mCherry in aIP-derived neurons (P21-35). Diagram of the experimental setup consisting of whole-cell voltage-clamp recordings of dSPNs and iSPNs while stimulating aIP-derived neurons with a 473 nm wide-field LED (right). Immunohistochemical staining (bottom, left) of two recorded and labeled SPNs as revealed with streptavidin-405 (in blue) in proximity to an aIP-derived ChR2-mCherry expressing SPN (in red). Inset: example current traces of evoked pharmacologically isolated IPSCs in response to brief light pulses. (**C**) aIP-derived striatal neurons exhibited a slight but significantly higher incidence of connecting to dSPNs (53%), but optical activation of aIP-derived neurons resulted in similar amplitude postsynaptic IPSCs in both dSPNs and iSPNs. (**D**) Plot of IPSC amplitude across developmental age for both E15.5 progenitor derived SPNs (black) and unlabeled SPNs (grey; unknown birthdate or progenitor origin). Dashed line is a linear fit to data suggesting that the increased strength of connections between SPNs of matched neurogenic stages is retained across the postnatal developmental time investigated.

Supplemental Tables

	aIP derived	OP derived	p-value	Unlabeled	ChR2+ aIP derived
Resting membrane potential (mV)	-78.47 ± 1.06	-80.20 ± 0.85	0.232	-80.58 ± 0.94	-79.61 ± 1.77
Input resistance (MΩ)	103.57 ± 3.92	97.22 ± 3.62	0.154	95.42 ± 5.36	101.43 ± 8.32
Membrane time constant (ms)	3.41 ± 0.22	2.72 ± 0.15	**0.009**	2.64 ± 0.17	3.45 ± 0.48
Spike threshold (mV)	-39.64 ± 0.82	-37.11 ± 0.83	**0.039**	-36.92 ± 1.24	-38.23 ± 0.92
Spike rate (500pA) (Hz)	38.61 ± 3.33	38.92 ± 3.61	0.953	36.53 ± 2.79	35.19 ± 6.97
Spike rate (400pA) (Hz)	35.71 ± 2.61	36.65 ± 3.01	0.824	34.22 ± 2.21	32.79 ± 4.45
Spike rate (300pA) (Hz)	31.68 ± 2.10	32.03 ± 2.51	0.919	29.00 ± 2.32	31.83 ± 2.65
Spike rate (200pA) (Hz)	23.40 ± 2.24	20.44 ± 2.10	0.348	22.39 ± 1.98	22.60 ± 3.02
Spike rate (100pA) (Hz)	12.81 ± 1.55	15.45 ± 3.40	0.440	6.56 ± 1.24	8.50 ± 1.70
First ISI (ms)	19.14 ± 1.57	20.57 ± 1.41	0.506	21.62 ± 1.79	19.85 ± 2.37
Second ISI (ms)	20.75 ± 1.38	22.00 ± 1.54	0.565	21.93 ± 1.35	19.44 ± 2.01
Third ISI (ms)	26.37 ± 1.79	26.62 ± 2.07	0.932	26.63 ± 1.73	26.32 ± 3.40
Fourth ISI (ms)	28.90 ± 1.60	27.57 ± 1.64	0.576	28.52 ± 1.70	25.82 ± 2.54
First spike amplitude (mV)	80.40 ± 1.61	77.07 ± 1.88	0.207	84.45 ± 2.47	75.03 ± 2.92
Second spike amplitude (mV)	64.34 ± 2.28	64.52 ± 2.16	0.954	72.45 ± 2.41	59.04 ± 3.41
First spike duration (ms)	1.83 ± 0.09	1.91 ± 0.13	0.633	1.74 ± 0.07	1.70 ± 0.15
Second spike duration (ms)	2.62 ± 0.16	2.53 ± 0.12	0.639	2.38 ± 0.11	2.63 ± 0.29

Data are given as mean ± SEM, statistical comparsions by *t*-test or Mann-Whitney U test

Table S1: Intrinsic membrane properties of striatal SPNs. Related to Figures 2 and 4.

DRAFT

	aIP derived	OP derived	p-value	Unlabeled
Soma surface area (μm^2)	482.27 ± 24.35	629.20 ± 51.60	**0.02**	671.47 ± 134.45
Number of Dendrites	6.82 ± 0.62	6.07 ± 0.33	0.08	5.38 ± 0.31
Total Dendritic Length (µm)	2177.73 ± 232.39	2346.70 ± 148.97	0.53	2162.70 ± 120.75
Average Dendrite Length (µm)	324.76 ± 24.92	398.20 ± 29.41	**0.03**	421.24 ± 26.54
Number of Branch Points	19.55 ± 1.65	16.20 ± 1.40	**0.05**	15.35 ± 0.88

Data are given as mean ± SEM, statistical comparsions by *t*-test or Mann-Whitney U test

DRAFT

Table S2: Morphological properties of striatal SPNs. Related to Figure 2.

Medial prefrontal cortex	aIP derived	OP derived	p-value	Unlabeled
Amplitude (mV)	3.77 ± 1.00	1.46 ± 0.59	**4.75E-2**	2.80 ± 0.60
Duration (ms)	161.83 ± 31.50	143.56 ± 44.30	0.46	125.92 ± 18.81
Rise time (ms)	6.64 ± 0.75	4.35 ± 1.01	0.68	6.46 ± 0.85
Decay time (ms)	75.28 ± 14.74	68.68 ± 22.09	0.33	57.08 ± 8.82
**Visual cortex**				
Amplitude (mV)	0.67 ± 0.27	3.54 ± 0.96	**1.03E-4**	2.80 ± 0.54
Duration (ms)	135.40 ± 21.15	155.53 ± 13.32	0.48	167.14 ± 19.85
Rise time (ms)	6.29 ± 1.08	6.22 ± 0.72	0.72	8.14 ± 0.62
Decay time (ms)	59.90 ± 10.33	72.55 ± 5.86	0.23	73.43 ± 9.88

Data are given as mean ± SEM, statistical comparisons by *t*-test.

Table S3: Synaptic EPSP properties to ChR2-mediated activation of cortical afferents (combined paired and unpaired recordings). Related to Figure 3.

	aIP derived to aIP derived	aIP derived to OP derived	p-value	aIP derived to dSPN	aIP derived to iSPN	p-value	aIP derived to Unlabeled
Amplitude (pA)	55.05 ± 15.29	48.70 ± 16.95	0.74	25.48 ± 9.28	25.55 ± 10.03	0.71	31.37 ± 12.14
Duration (ms)	137.50 ± 20.46	157.69 ± 25.18	0.50	111.81 ± 11.78	97.44 ± 15.87	0.46	117.46 ± 21.86
Rise time (ms)	6.85 ± 1.16	6.76 ± 1.03	0.77	4.37 ± 0.67	5.06 ± 1.41	0.90	6.79 ± 1.68
Decay time (ms)	64.28 ± 9.70	77.81 ± 11.42	0.25	57.06 ± 7.91	46.06 ± 9.43	0.38	53.74 ± 10.19

Data are given as mean ± SEM, statistical comparisons by Mann-Whitney U test

Table S4: Synaptic IPSC properties from aIP derived SPNs. Related to Figure 4 and Supplemental Figure 4.

DRAFT
