# Supplementary material for: Diversity in striatal synaptic circuits arises from distinct embryonic progenitor pools in the ventral telencephalon
Source: Cell Rep. 2021 Apr 27;35(4):109041. doi: 10.1016/j.celrep.2021.109041 (PMC8097690; doi:10.1016/j.celrep.2021.109041)
Supplement: Document S2. Article plus supplemental information [file mmc2.pdf]

Cell Reports

Report

Diversity in striatal synaptic circuits arises from distinct embryonic progenitor pools in the ventral telencephalon

Graphical abstract

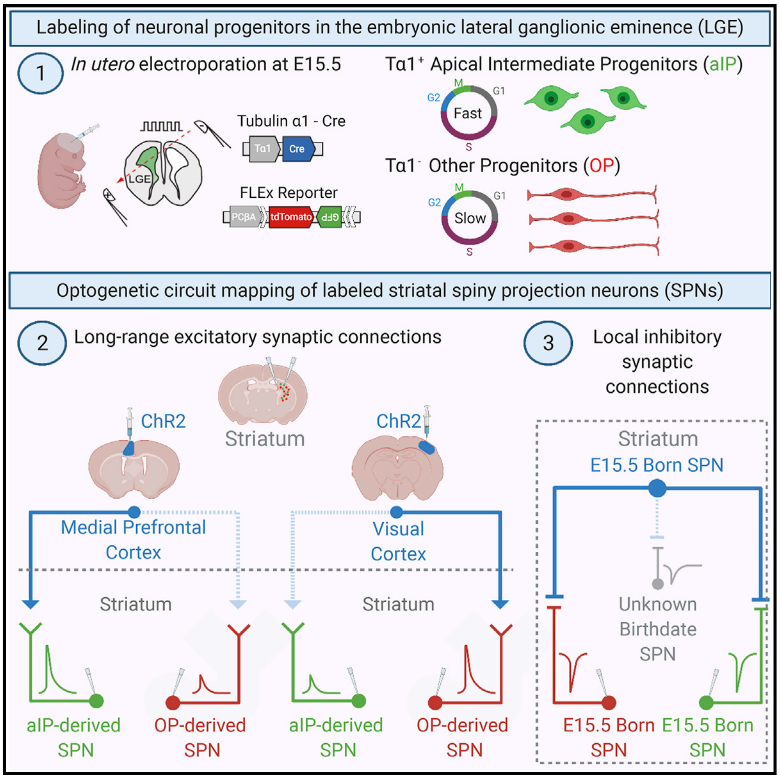

Highlights

- The Tα1 promoter distinguishes two embryonic progenitor pools in the LGE
- Both pools generate intermixed spiny projection neurons in dorsomedial striatum
- Excitatory cortical inputs are biased toward SPNs of different embryonic origin
- Neurogenic stage rather impacts local inhibitory connections among SPNs

Authors

Fran van Heusden, Anežka Macey-Dare, Jack Gordon, Rohan Krajeski, Andrew Sharott, Tommas Ellender

Correspondence

tommas.ellender@pharm.ox.ac.uk

In brief

van Heusden et al. demonstrate significant roles for distinct embryonic progenitor pools in the formation of postnatal striatal synaptic circuits. Diverse progenitor pools generate striatal spiny projection neurons with similar intrinsic properties and convey biases in their excitatory cortical synaptic inputs, whereas birthdate affects strength of local inhibitory synaptic inputs.

van Heusden et al., 2021, Cell Reports *35*, 109041
April 27, 2021 © 2021 The Author(s).
https://doi.org/10.1016/j.celrep.2021.109041

CellPress

Cell Reports

CellPress
OPEN ACCESS

Report

Diversity in striatal synaptic circuits arises from distinct embryonic progenitor pools in the ventral telencephalon

Fran van Heusden,[1,3] Anežka Macey-Dare,[1,3] Jack Gordon,[1] Rohan Krajeski,[1] Andrew Sharott,[2] and Tommas Ellender[1,4,*]
[1]Department of Pharmacology, University of Oxford, Oxford OX1 3QT, UK
[2]MRC BNDU, University of Oxford, Oxford OX1 3TH, UK
[3]These authors contributed equally
[4]Lead contact
*Correspondence: tommas.ellender@pharm.ox.ac.uk
https://doi.org/10.1016/j.celrep.2021.109041

SUMMARY

Synaptic circuits in the brain are precisely organized, but the processes that govern this precision are poorly understood. Here, we explore how distinct embryonic neural progenitor pools in the lateral ganglionic eminence contribute to neuronal diversity and synaptic circuit connectivity in the mouse striatum. *In utero* labeling of Tα1-expressing apical intermediate progenitors (aIP), as well as other progenitors (OP), reveals that both progenitors generate direct and indirect pathway spiny projection neurons (SPNs) with similar electrophysiological and anatomical properties and are intermingled in medial striatum. Subsequent optogenetic circuit-mapping experiments demonstrate that progenitor origin significantly impacts long-range excitatory input strength, with medial prefrontal cortex preferentially driving aIP-derived SPNs and visual cortex preferentially driving OP-derived SPNs. In contrast, the strength of local inhibitory inputs among SPNs is controlled by birthdate rather than progenitor origin. Combined, these results demonstrate distinct roles for embryonic progenitor origin in shaping neuronal and circuit properties of the postnatal striatum.

INTRODUCTION

A key question in neuroscience is how neuronal identity and precise synaptic connectivity within neuronal circuits emerges and what critically guides this process. Studies in the dorsal telencephalon have shown important and distinct roles for individual embryonic progenitors (Yu et al., 2009; Yu et al., 2012; Cadwell et al., 2020) or distinct progenitor pools (Tyler et al., 2015; Ellender et al., 2019, in shaping neuronal identity and synaptic connectivity in postnatal circuits. However, far less is known about how embryonic progenitors in the ventral telencephalon contribute to cellular diversity and neural circuitry in ventral structures, such as the basal ganglia and striatum. These neural progenitors are found in ganglionic eminences; transitory structures that generate most interneurons in the brain, and spiny projection neurons (SPNs) of the striatum (Halliday and Cepko, 1992; Wonders and Anderson, 2006; Wamsley and Fishell, 2017; Tinterri et al., 2018). Here, we focused on the neural progenitors of the lateral ganglionic eminence (LGE) that generate striatal SPNs during the latter part of embryogenesis (van der Kooy and Fishell, 1987) and include radial glial cells, short neural precursors, and subapical progenitors, among others (Olsson et al., 1998; Stenman et al., 2003; Mason et al., 2005; Pilz et al., 2013; Kelly et al., 2018), many of which have been characterized also in cortical proliferative zones (Noctor et al., 2001, 2004; Gal et al., 2006; Kowalczyk et al., 2009; Stancik et al., 2010; Shitamukai et al., 2011; Wang et al., 2011; Franco and Müller, 2013; Taverna et al., 2014).

The striatum is the main input nucleus of the basal ganglia and is key in the regulation of motor and cognitive function (Graybiel et al., 1994; Grillner et al., 2005; Yin and Knowlton, 2006; Kravitz et al., 2010; Cui et al., 2013; Tecuapetla et al., 2016). It can be split into large functional domains, defined by anatomical subregion and/or neurochemical expression, which are thought to shape parallel functional pathways through the basal ganglia (Graybiel and Ragsdale, 1978; Alexander et al., 1986; Graybiel, 1990; Haber, 2008; Pan et al., 2010; Oh et al., 2014; Hintiryan et al., 2016; Hunnicutt et al., 2016; McGregor et al., 2019; Lee et al., 2020). All domains contain a mixture of direct pathway SPNs (dSPNs) and indirect pathway SPNs (iSPNs), the striatal GABAergic projection neurons with distinct long-range outputs (Day et al., 2008; Gertler et al., 2008) that process and integrate excitatory inputs from distinct brain regions and interact via lateral inhibitory connections in the striatum (Taverna et al., 2008; Planert et al., 2010; Chuhma et al., 2011; Burke et al., 2017; Krajeski et al., 2019). It has been shown that long-range glutamatergic synapses from different cortical regions can converge onto single SPNs (Reig and Silberberg, 2014) or diverge and form biased synaptic connections on either dSPNs or iSPNs (Wall et al., 2013; Johansson and Silberberg, 2020). Local inhibitory connections between SPNs also exhibit biases in that iSPNs form more frequent and stronger synaptic

This is an open access article under the CC BY license (http://creativecommons.org/licenses/by/4.0/).

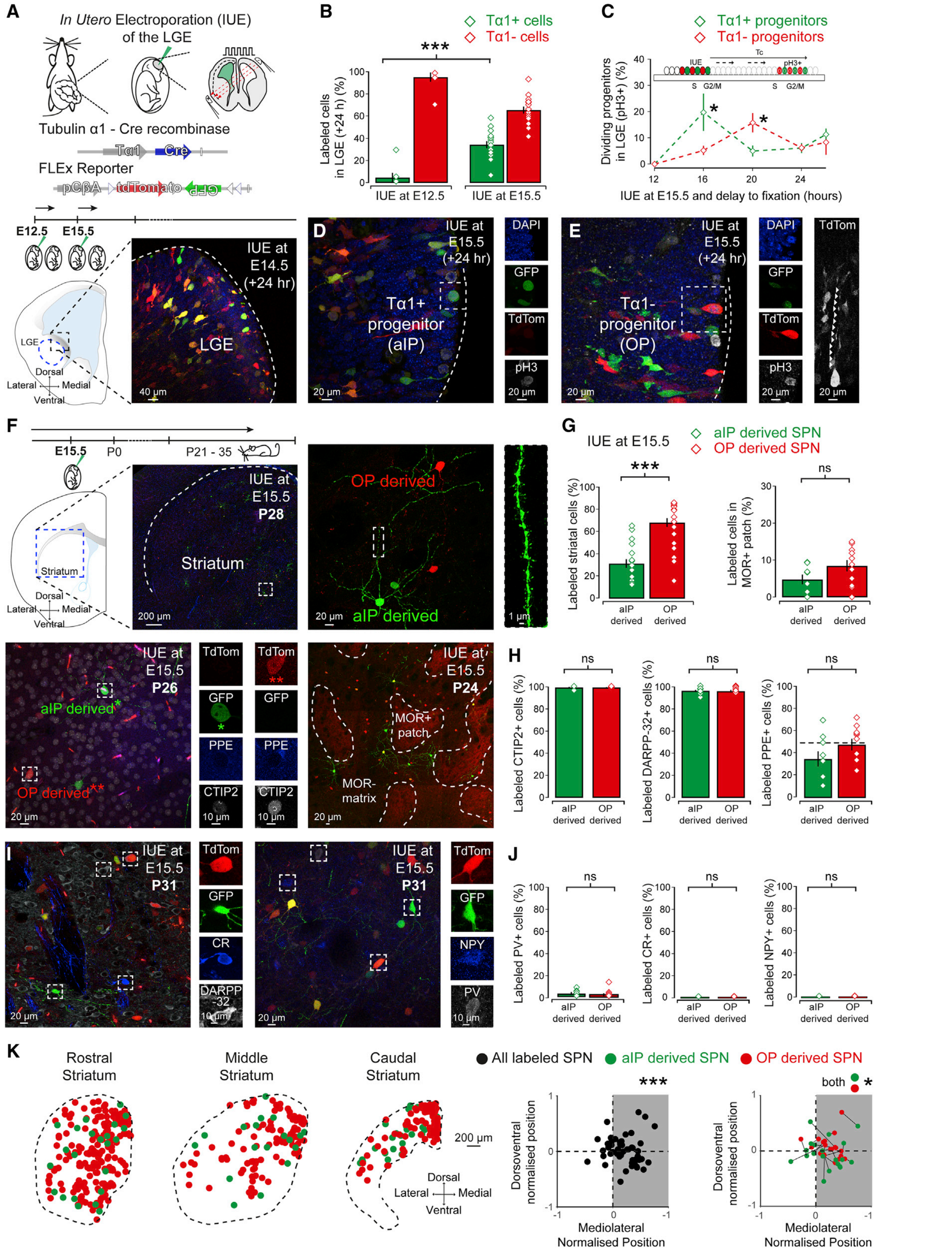

(legend on next page)

CellPress
OPEN ACCESS

connections (Taverna et al., 2008; Planert et al., 2010; Cepeda et al., 2013; Krajeski et al., 2019). Considering that young SPNs exhibit complex migratory pathways and intermix during development (Tinterri et al., 2018), how do precise striatal synaptic circuits develop? Do distinct SPN progenitor lineages and/or birthdates important for striosome/matrix formation (Kelly et al., 2018; Matsushima and Graybiel, 2020) also shape the development of other striatal circuits? Are connectivity rules based on embryonic progenitor origin as described in cortex (e.g., "out-of-class" local connectivity biases [Ellender et al., 2019]) also found in the striatum? To address such questions, we used *in utero* electroporation (IUE) in mice to fluorescently pulse-label two pools of LGE embryonic progenitors, distinguished by their differential expression of the tubulin alpha1 (Tα1) gene (Gal et al., 2006; Stancik et al., 2010), which was previously used to selectively label short neural precursors in the cortical ventricular zone (VZ) (Gal et al., 2006; Stancik et al., 2010; Tyler and Haydar, 2013; Ellender et al., 2019), in combination with electrophysiological, anatomical, and optogenetic circuit-mapping studies to explore how SPN embryonic origin shapes postnatal striatal circuits.

RESULTS

The LGE contains distinct embryonic progenitor pools that generate dSPNs and iSPNs

The embryonic LGE contains a diverse group of neural progenitors (Pilz et al., 2013; Kelly et al., 2018). To genetically distinguish between different progenitor pools, two DNA constructs were electroporated: a Tα1-Cre construct where Cre recombinase is controlled by part of the Tα1 promoter (Stancik et al., 2010) and a CβA-FLEx reporter construct incorporating a flexible excision (FLEx) cassette where Cre recombination permanently switches expression from the fluorescent protein TdTomato to GFP (Franco et al., 2012; Figure 1A). 24 h post IUE, allowing for recombination, the LGE contained both GFP and TdTomato (TdTom) expressing cells, consisting of Tα1-expressing (Tα1$^+$) and non-Tα1-expressing (Tα1$^-$) neural progenitors and young migrating neurons (Figure 1A). Throughout the paper, "n/n" refers to number of cells and mice, respectively. Initial IUE was performed at embryonic day (E)12.5 or E15.5 and greater numbers of Tα1$^+$/GFP$^+$ cells were observed after IUE at E15.5 (E12.5: 4.8% ± 4.1% and E15.5: 34.5% ± 3.0%, Mann-Whitney, p = 0.002, n = 250/7, and 1,204/17) (Figure 1B), suggesting Tα1-expressing progenitors form a large proliferating population during later-stage neurogenesis. Hence, we focused on E15.5 for subsequent studies. We found that Tα1$^+$/GFP$^+$ and Tα1$^-$/TdTom$^+$ progenitor properties diverged in several ways. First, Tα1$^+$/GFP$^+$ progenitors returned to G1/S phase faster, as assessed through labeling with the mitotic marker phospho-histone H3 (pH3) (at 16 h; Tα1$^+$/GFP$^+$: 19.85% ± 7.15% and Tα1$^-$/TdTom$^+$: 5.18% ± 1.72%, t test, p = 0.025, n = 318/6) (Figure 1C), suggesting faster cell-cycle kinetics. Second, during division, many Tα1$^+$/GFP$^+$ progenitors had a short, rounded morphology (Figures 1D and S1A), whereas many Tα1$^-$/TdTom$^+$ progenitors retained their basal process (Tα1$^+$/GFP$^+$ + process: 20.6% ± 4.7% and Tα1$^-$/TdTom$^+$ + process: 35.9% ± 7.8%, Wilcoxon signed rank, p = 0.029, n = 961/15) (Figures 1E, S1B, and S1C). These distinct progenitors and their progeny were found intermingled throughout the VZ in all LGE subregions (Stenman et al., 2003; Flames et al., 2007; Tucker et al., 2008; Xu et al., 2018) and showed similar lateral and horizontal migration 24 h post IUE (Halliday and Cepko, 1992; Reid and Walsh, 2002; Tinterri et al., 2018), although Tα1$^+$/GFP$^+$-labeled cells were found slightly further from the ventricle (Tα1$^+$/GFP$^+$: 66.24 ± 3.57 μm and Tα1$^-$/TdTom$^+$: 56.45 ± 2.90 μm, t test, p = 0.0055, n = 1,434/30) (Figures S1D and S1E), possibly reflecting faster cell-cycle kinetics. Control experiments revealed recombination occurred during embryonic development and accurately reflected the promoter driving Cre expression (Figure S2).

Together, these studies show that our labeling delineates two apically dividing LGE progenitor pools: a Tα1-expressing pool similar in morphology and cell-cycle kinetics to short neural precursors and subapical progenitors (Pilz et al., 2013; Kelly et al., 2018; Ellender et al., 2019) and a non-Tα1-expressing pool similar to radial glial cells (Pilz et al., 2013; Kelly et al., 2018). As many subapical progenitors derive from short neural precursors (Pilz et al., 2013) and are therefore closely lineally related, as both divide in the apical aspects of the LGE proliferative zone, and to conform to nomenclature of similar cortical (Tyler and Haydar, 2013; Ellender et al., 2019) and LGE embryonic progenitors (Kelly et al., 2018), we refer to the GFP$^+$ Tα1-expressing progenitors collectively as apical intermediate progenitors (aIP). The TdTom$^+$ non-Tα1-expressing progenitors

Figure 1. Diverse LGE embryonic progenitor pools generate both dSPNs and iSPNs found intermixed in medial striatum

(A) IUE of Tα1-Cre and FLEx reporter plasmids labeled Tα1-expressing (Tα1$^+$/GFP$^+$) and non-Tα1-expressing (Tα1$^-$/ TdTom$^+$) LGE progenitors.

(B) Tα1$^+$/GFP$^+$ progenitors formed a large population during later embryonic stages.

(C) Tα1$^+$/GFP$^+$ progenitors returned to a mitotic phase (M) earlier than Tα1$^-$/TdTom$^+$ progenitors (at 20 h post E15.5 IUE; Tα1$^+$/GFP$^+$: 4.98% ± 2.11% and Tα1$^-$/TdTom$^+$: 15.81% ± 3.63%, t test, p = 0.036, n = 418/8). pH3 was used as a mitotic marker.

(D) Mitotic Tα1$^+$/GFP$^+$ progenitors at the ventricle often had a rounded morphology lacking a basal process.

(E) In contrast, many mitotic Tα1$^-$/TdTom$^+$ progenitors at the ventricle retained a basal process during division.

(F) The postnatal (P21–P35) striatum of IUE mice contained both aIP- and OP-derived neurons with spiny dendrites (top, right) and expressed molecular markers characteristic of striatal SPNs (bottom, left). aIP- and OP-derived neurons were found both in MOR$^+$ patches (red) and MOR$^-$ matrix compartments (bottom right).

(G) Relative numbers of aIP- and OP-derived neurons reflected the embryonic ratio of progenitors and were found in equal numbers within MOR$^+$ patches.

(H) Virtually all aIP- and OP-derived neurons expressed the SPN markers CTIP2 and DARPP-32 and consisted of both PPE$^-$/dSPNs and PPE$^+$/iSPNs.

(I) Labeling for the markers DARPP-32 and CR (left) or PV and NPY (right).

(J) Little to no co-expression was seen in aIP- or OP-derived neurons with interneuronal markers PV, NPY, or CR.

(K) aIP- and OP-derived SPNs were found throughout striatum with on average a significant medial bias. Representative distribution of aIP- and OP-derived SPNs in an IUE brain (left). Scatterplot of dorsoventral and mediolateral position of all labeled SPNs (middle) and aIP- and OP-derived SPNs (right). Data are represented as mean ± SEM.

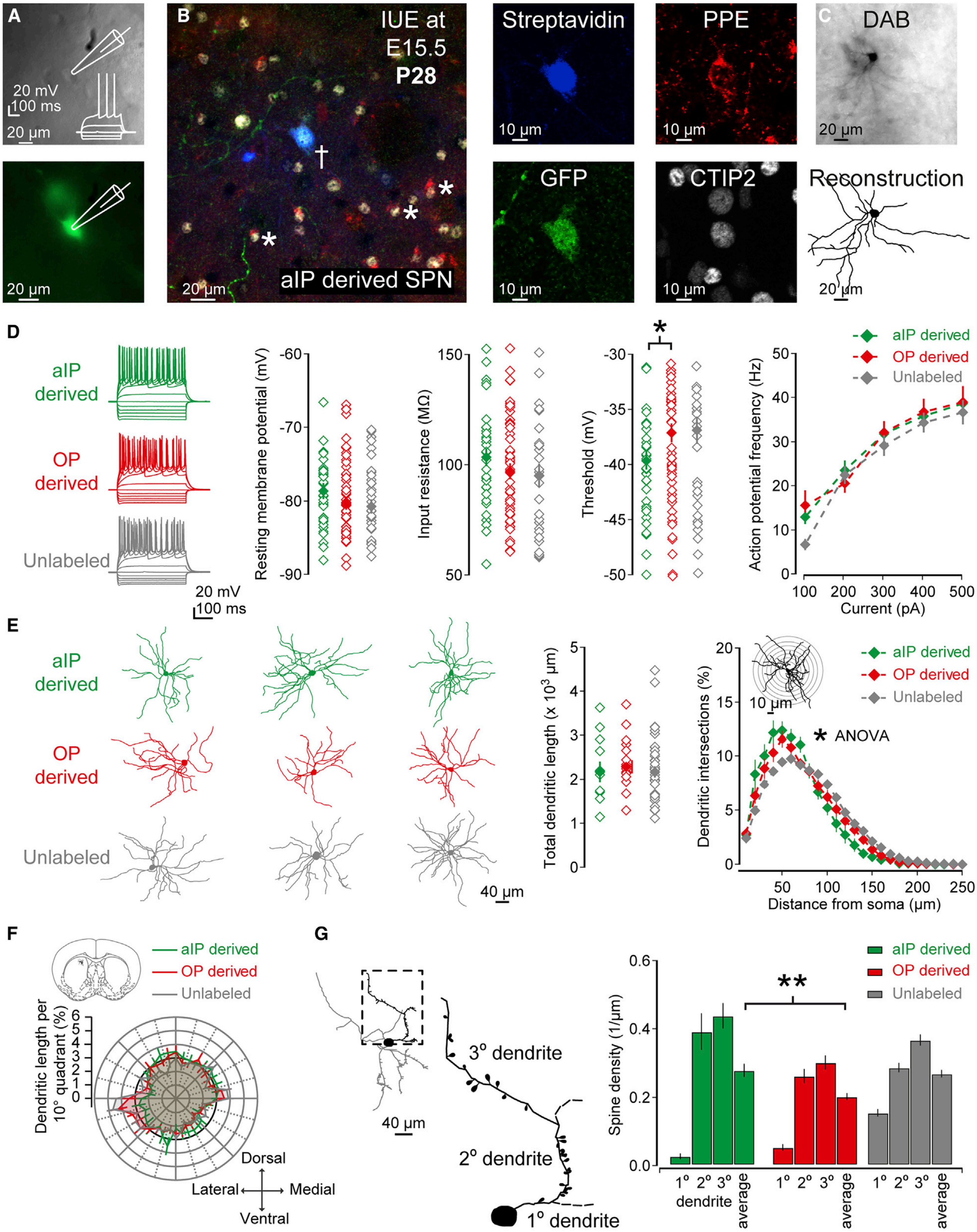

(legend on next page)

are referred to as other progenitors (OP), likely comprising a more heterogeneous population.

Electroporated embryos were left to develop normally, and postnatal brains (postnatal day [P] P21–P35) revealed many GFP$^+$- and TdTom$^+$-labeled neurons interspersed throughout the striatum, with a few also in the olfactory bulb (Stenman et al., 2003; Figure S3D). Because the GFP$^+$ neurons were born from aIP progenitors, they are referred to as aIP-derived, whereas the TdTom$^+$ neurons generated from OP progenitors are referred to as OP-derived (Figure 1F). Labeled neurons exhibited the radial morphology of striatal SPNs with spiny dendrites and expressed many SPN molecular markers (Figure 1F). Quantification of progenitor-derived striatal neurons (a total of >5,000 neurons in sections from 24 brains) revealed that the ratio of aIP- and OP-derived neurons reflected that of aIP and OP progenitors at E15.5 (aIP-derived: 31.6% ± 3.4% and OP-derived: 68.4% ± 3.4%, t test, p = 0.00014, n = 24 brains) (Figures 1G and S2G). Labeling for striatal markers showed that both could be found in μ-opioid receptor (MOR)-rich patches but were predominantly located in the MOR-poor matrix compartments (aIP/MOR$^+$: 4.8% ± 1.3% and OP/MOR$^+$: 7.8% ± 1.6%, n = 12 mice) (Figures 1F and 1G). Virtually all labeled neurons co-localized with the SPN markers CTIP2 (aIP/CTIP2$^+$: 99.6% ± 0.4% and OP/CTIP2$^+$: 99.4% ± 0.3%, n = 9 mice) (Figures 1F and 1H) and DARPP-32 (aIP/DARPP-32$^+$: 96.6% ± 0.9% and OP/DARPP-32$^+$: 96.3% ± 1.0%, n = 13 mice) (Figures 1H and 1I), thus indicating that most aIP- and OP-derived neurons are SPNs (Arlotta et al., 2008). Moreover, it showed that roughly half of aIP- and OP-derived SPNs expressed the iSPN marker pre-proenkephalin (PPE) (Gerfen et al., 1990) (aIP/PPE$^+$: 35.2% ± 7.0% and OP/PPE$^+$: 48.2% ± 5.3%, n = 9 mice) (Figures 1F and 1H). We did not find evidence for significant co-expression in aIP- and OP-derived SPNs with the interneuronal markers parvalbumin (aIP/PV$^+$: 3.2% ± 1.2% and OP/PV$^+$: 2.1% ± 1.2%, n = 13 mice), neuropeptide Y (aIP/NPY$^+$: 0.0% ± 0.0% and OP/NPY$^+$: 0.0% ± 0.0%, n = 13 mice), or calretinin (aIP/CR$^+$: 0.0% ± 0.0% and OP/CR$^+$: 0.03% ± 0.03%, n = 13 mice) (Figures 1I and 1J). Combined, these data suggest that aIP and OP LGE progenitors generate striatal SPNs consisting of both dSPNs and iSPNs found in both patch and matrix compartments.

Finally, we performed unbiased stereological analysis of the spatial distribution of aIP- and OP-derived CTIP2$^+$ SPNs in striatum (Figures 1G and S3A). Progenitor-derived SPNs were found in all striatal regions (rostral, middle, and caudal striatum), however, the overall normalized position was biased toward the medial aspects (Wilcoxon signed rank, p = 0.000005, n = 2,246/15) (Figure 1H) but not the dorsal or ventral aspects (p > 0.05), with medial preference seen for both aIP- and OP-derived SPNs (aIP-derived: 60% and OP-derived: 45% of sections, p < 0.016) (Figure 1H). We also found the relative aIP- and OP-derived SPN densities were similar between regions (Figure S3B) but their spread differed significantly with OP-derived SPNs exhibiting a greater spread (Figure S3C), suggesting different migration patterns (Halliday and Cepko, 1992; Tan and Breen, 1993; Reid and Walsh, 2002; Tinterri et al., 2018).

aIP- and OP-derived SPNs have similar electrical and morphological properties

We next explored the electrophysiological and morphological properties of aIP- and OP-derived SPNs in dorsomedial striatum (DMS), using whole-cell patch-clamp recordings and post hoc reconstruction of recorded SPNs (Figures 2A–2C). A total of 39 aIP-derived, 57 OP-derived, and 33 unlabeled (control) SPNs (n = 54 mice) were recorded to investigate various electrophysiological properties (Figure 2D; Table S1). This revealed that aIP- and OP-derived neurons had remarkably similar properties consistent with those of SPNs (Figure 2D; Table S1; Day et al., 2008; Gertler et al., 2008; Krajeski et al., 2019) and only subtly differed in their action potential threshold (aIP: −39.64 ± 0.82 mV, OP: −37.11 ± 0.83 mV and unlabeled: −36.92 ± 1.24 mV; Mann-Whitney, p = 0.039) (Figure 2E) and membrane time constant (aIP: 3.41 ± 0.22 ms, OP: 2.72 ± 0.15 ms and unlabeled: 2.64 ± 0.17 ms; Mann-Whitney, p = 0.009) (Table S1), suggesting similar responsiveness to synaptic inputs.

A total of 13 aIP-derived, 28 OP-derived, and 30 unlabeled SPNs (n = 29 mice) were processed further with DAB for reconstruction of dendritic arbors and/or spine counting (Figures 2E–2G; Table S2). This revealed that aIP-derived SPN soma were subtly smaller, consistent with their longer membrane time constants (Table S2). Although total dendritic length was similar (aIP: 2,184.7 ± 228.8 μm, OP: 2,299.3 ± 147.2 μm and unlabeled: 2,182.6 ± 129.3 μm; Kruskal-Wallis, p = 0.626) (Figure 2E) and dendrites were radially oriented (Figure 2F), the aIP-derived SPNs had greater dendritic complexity close to the soma (ANOVA, p = 0.048, n = 11/6 and 16/12) (Figure 2E) and a greater overall number of branch points (aIP: 19.55 ± 1.65 and OP: 16.20 ± 1.40, Mann-Whitney, p = 0.05) (Table S2). Dendritic spine counts revealed that aIP-derived SPNs exhibited higher average spine densities than OP-derived SPNs (aIP: 0.28 ± 0.02 spines/μm and OP: 0.20 ± 0.01 spines/μm, t test, p = 0.0058, n = 13/8

Figure 2. Embryonic progenitor pool conveys subtle differences in SPN neuronal excitability and morphology

(A) Electrophysiological properties of progenitor derived SPNs (P21–P35) were assessed using whole-cell patch-clamp recordings in brain slices. Inset: SPN response to current steps.

(B) Recorded SPNs (†) were labeled with biocytin and revealed in fixed tissue using immunofluorescence, and some tested for expression of CTIP2 and PPE. Neighboring CTIP2$^+$/PPE$^+$ neurons are also shown (*).

(C) Recorded SPNs were processed with DAB allowing for reconstruction and spine counting.

(D) Current steps were used to characterize the electrophysiological properties of SPNs. Note the small but significant difference in spike threshold between aIP- and OP-derived SPNs.

(E) Reconstructed SPN examples (left). Whereas no differences in total dendritic length were found (middle), aIP-derived SPNs showed subtle but significant greater proximal dendritic complexity (right).

(F) Polarity analysis revealed that all SPNs exhibited a similar radial morphology.

(G) Dendritic spine quantification revealed that aIP-derived SPNs exhibited a higher average density of spines relative to OP-derived SPNs. Data are represented as mean ± SEM.

and 28/18) (Figure 2G). Combined, these results suggest that aIP- and OP-derived neurons show remarkably similar electrical and morphological properties consistent with SPNs and only subtle differences in excitability and morphology.

Embryonic progenitor origin generates biases in long-range cortical excitatory synaptic connections onto SPNs

Biases in long-range excitatory synaptic connectivity in the cortex can arise from distinct embryonic progenitor pools (Ellender et al., 2018, 2019). Next, we explored whether aIP- and OP-derived SPNs differentially sample excitatory input coming from distinct cortical regions. Because the progenitor-derived SPNs were preferentially located in the medial aspects, we focused on two dominant cortical regions projecting to DMS (Pan et al., 2010; Oh et al., 2014; Guo et al., 2015; Hunnicutt et al., 2016). This striatal area is thought to integrate heterogeneous, multimodal inputs coming from medial prefrontal cortex (mPFC) (Laubach et al., 2018) and visual cortex (VC) (Khibnik et al., 2014), with both regions directly innervating SPNs (Wall et al., 2013; Khibnik et al., 2014; Reig and Silberberg, 2014; Loewke et al., 2020). In young mice that had undergone IUE to label aIP- and OP-derived SPNs, these cortical regions were targeted with localized injections (150 nL) of AAV1-CAMKII-ChR2-GFP viral particles (Hammad et al., 2015; Arruda-Carvalho et al., 2017) to transfect excitatory cortical neurons with ChR2-GFP (Figure 3A). Three weeks later and onward, simultaneous paired current-clamp recordings of aIP- and OP-derived striatal SPNs were combined with optical activation of cortical afferents (Figure 3A). This showed SPNs were directly innervated by afferents from both mPFC and VC, and synapses exhibited pronounced short-term depression in response to optical stimulation trains, greatest for mPFC inputs (Figure 3B), consistent with previous work and accurate cortical region targeting (Hunnicutt et al., 2016; Lu et al., 2019). Interestingly, we found a clear dissociation in the strength of excitatory innervation of striatal SPNs derived from the different pools. When axons from mPFC were activated, we saw a stronger excitatory drive to aIP-derived SPNs than to simultaneously recorded OP-derived SPNs, captured as an output bias toward aIP-derived SPNs of 0.73 ± 0.04, where 0.5 represents equal output ($p = 0.00032$, t test, $n = 11/7$) (Figure 3C). Conversely, VC inputs provided a stronger excitatory drive to OP-derived SPNs than to neighboring aIP-derived SPNs (bias toward OP of 0.33 ± 0.05; $p = 0.0054$, t test, $n = 10/9$) (Figure 3D). Paired recordings from unlabeled SPNs in the same sections showed no systematic differences in inputs from either mPFC (0.54 ± 0.08; $p = 0.84$, t test, $n = 19/6$) (Figure 3E) or VC (0.53 ± 0.07; $p = 0.67$, t test, $n = 9/8$) (Figure 3F). Other synaptic response properties did not differ between recorded SPN pairs (Table S3). Together, these results reveal that embryonic progenitor origin strongly biases the long-range inputs from different cortical regions onto striatal SPNs.

Local inhibitory synaptic connections are stronger among SPNs with similar birthdates

We next studied whether embryonic progenitor origin also impacts local inhibitory synaptic connections between SPNs (Taverna et al., 2008; Planert et al., 2010; Burke et al., 2017; Krajeski et al., 2019). Indeed, it was recently shown that cortical excitatory neurons prefer to make local synaptic connections with neurons derived from other embryonic progenitor pools (Ellender et al., 2019). To investigate this in striatum, we used optogenetic circuit-mapping approaches. First, we asked whether dSPNs and iSPNs sample synaptic inputs from aIP-derived SPNs. We performed IUE at E15.5 of Tα1-Cre and floxed-ChR2 plasmids (Saunders et al., 2012) in D1 or D2-GFP transgenic mice to selectively express the light-activatable channel ChR2 in aIP-derived SPNs and studied how dSPNs and iSPNs sample afferents coming from aIP-derived SPNs (Figure S4B). Whole-cell voltage-clamp recordings (at +30 mV) of both dSPNs and iSPNs were made in DMS and combined with brief blue light pulses to activate afferents coming from aIP-derived SPNs (Figure S4B). We found both dSPNs and iSPNs received synaptic inputs, and although evoked current amplitudes were similar (dSPN: 25.48 ± 9.28 pA, iSPN: 25.55 ± 10.03 pA, Mann-Whitney, $p = 0.71$, $n = 17/10$ and $7/5$) (Figure S4C; Table S4), the incidence of finding a connection from aIP-derived SPNs to dSPNs was higher than to iSPNs (dSPN: 17/33; 51.5% and iSPN: 7/28 connections; 25.0%; $p = 0.040$, Fisher's exact) (Figure S4C), suggesting preferential innervation of dSPNs.

We next studied how aIP- and OP-derived SPNs sample local GABAergic synaptic afferents coming from aIP-derived SPNs. We used IUE of Tα1-Cre, FLEx, and floxed-ChR2 plasmids to selectively express ChR2 in aIP-derived neurons and fluorescently label aIP- and OP-derived SPNs (Figure 4A). Brief blue light pulses generated action potentials in patched aIP-derived $ChR2^+$ SPNs (Figure 4B), and ChR2-expression did not seem to affect their intrinsic properties (Table S1). Simultaneous whole-cell voltage-clamp recordings from aIP- and OP-derived SPNs were combined with optical activation of afferents from aIP-derived SPNs, which generated prominent currents in recorded SPNs (Figure 4C). For aIP-derived $ChR2^+$ SPNs, this consisted of combined optical and synaptic currents (Figure 4D) so all recordings were made at +30 mV, close to the ChR2-mediated current reversal potential (Nagel et al., 2003; Berndt et al., 2011), and the GABA receptor-mediated synaptic current component was isolated by superfusing the $GABA_A$ receptor antagonist SR95531 (120 nM) (Figures 4D and S4A; STAR Methods; Stell and Mody, 2002). These data showed that both aIP- and OP-derived SPNs sample GABAergic afferents coming from aIP-derived SPNs, and both the incidence of synaptic connections (aIP: 47.5% and OP: 27.5%, $p = 0.11$, Fisher's exact) (Figure 3E) and the inhibitory postsynaptic current (IPSC) amplitudes (aIP: 55.05 ± 15.29 pA and OP: 48.70 ± 16.95 pA, Mann-Whitney, $p = 0.735$, $n = 19/11$ and $11/6$) (Figure 4E; Table S4) were similar. This suggests that embryonic progenitor origin does not significantly bias local synaptic connections between SPNs. However, when exploring how unlabeled SPNs (i.e., of unknown birthdate/origin) within the same sections sample inputs from aIP-derived SPNs, we found their postsynaptic IPSCs had smaller amplitudes than those in E15.5 pulse-labeled aIP- and OP-derived SPNs (E15.5-labeled SPNs: 52.72 ± 11.34 pA and unlabeled SPNs: 28.02 ± 6.42 pA, Mann-Whitney, $p = 0.018$, $n = 30/13$ and $42/20$) (Figure 4F), suggesting stronger synaptic connections among SPNs with similar birthdates. We

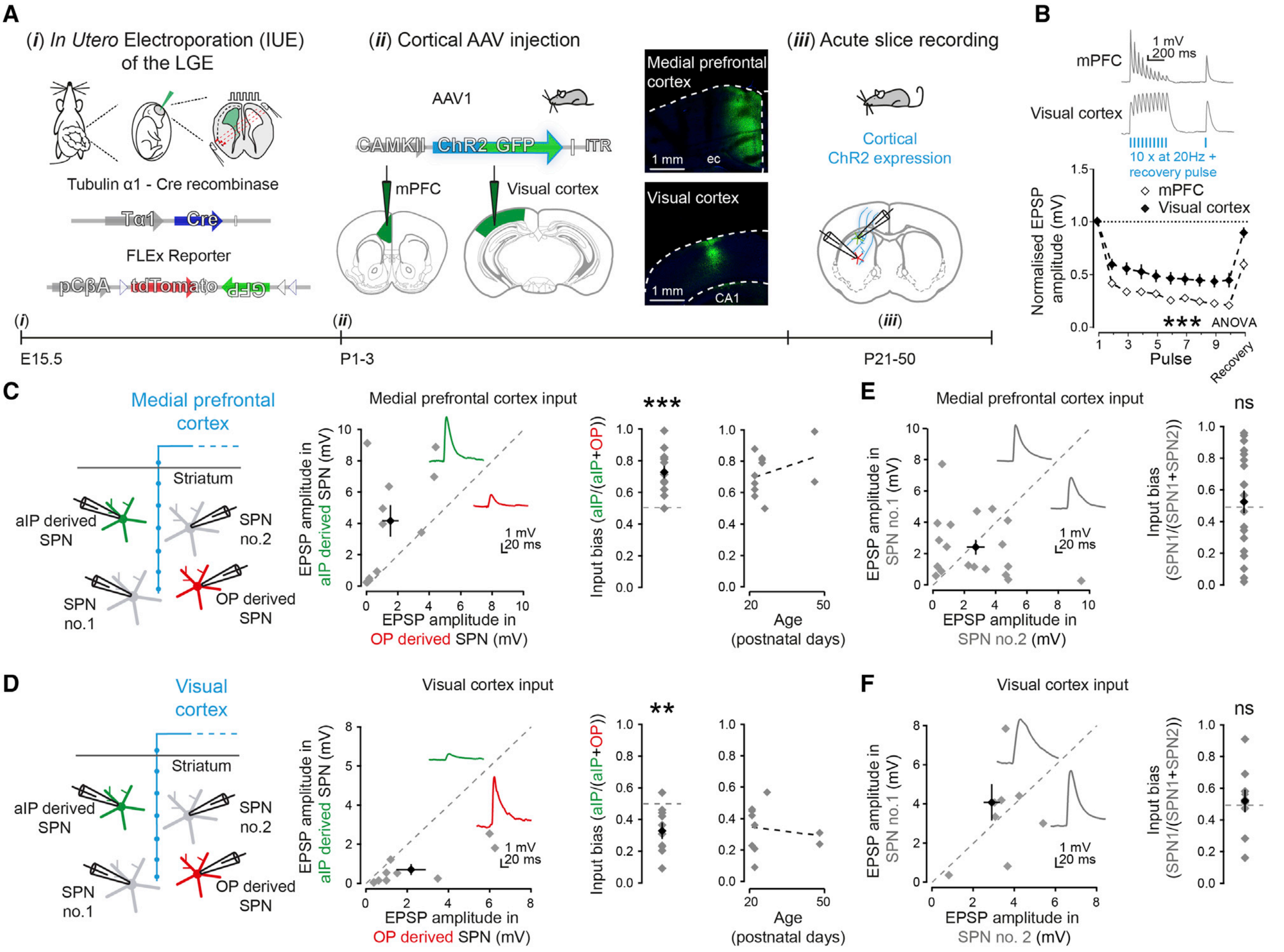

Figure 3. Differential innervation of aIP- or OP-derived SPNs by long-range excitatory synaptic inputs from mPFC and visual cortex (VC)

(A) IUE at E15.5 of Tα1-Cre and FLEx reporter plasmids labeled SPNs as a function of their progenitor of origin (*i*). Mice aged P1–P3 received a targeted injection of AAVs encoding CAMKII-ChR2 into either mPFC or VC. (*ii*). After 3+ weeks (P21–P50), dual whole-cell current-clamp recordings were made from neighboring aIP- and OP-derived SPNs and ChR2-expressing cortical fibers activated optically (*iii*).

(B) Short-term plasticity at synapses from mPFC or VC onto striatal SPNs differed (p = 1.45E−30, ANOVA, mPFC n = 29/6 and VC n = 15/6).

(C) aIP-derived SPNs had significantly stronger responses to activation of mPFC inputs than simultaneously recorded OP-derived SPNs (aIP: 4.24 ± 1.02 mV and OP: 1.57 ± 0.52 mV, n = 11 pairs/7). This difference is expressed as an input bias. Plot of observed input bias in relation to age (right).

(D) In contrast, aIP-derived SPNs had significantly weaker responses to VC inputs than simultaneously recorded OP-derived SPNs (aIP: 0.79 ± 0.27 mV and OP: 1.98 ± 0.71 mV, n = 10 pairs/9).

(E and F) Dual recordings of neighboring pairs of unlabeled SPNs did not show systematic differences in postsynaptic responses to (E) activation of mPFC afferents or (F) activation of VC afferents. Data are represented as mean ± SEM.

found this is not unique to aIP-derived SPNs because synaptic connections between labeled SPNs using CAG promoter-driven plasmids at E15.5 were also larger in amplitude than simultaneously recorded unlabeled SPNs of unknown birthdate (E15.5 $ChR2^+$ SPNs: 43.70 ± 13.07 pA, E15.5 $TdTom^+$ SPNs: 36.30 ± 14.40 pA and unlabeled SPNs: 19.31 ± 5.99 pA, E15.5 labeled versus unlabeled, Mann-Whitney, p = 0.015, n = 12/10, 9/7 and 21/7) (Figure 4G).

Together, these results demonstrate that aIP-derived SPNs integrate within local striatal circuits, and that the neurogenic stage (i.e., the time at which neurons are formed), rather than embryonic origin, contributes to the future local inhibitory connection strength among SPNs.

DISCUSSION

In summary, we combined *in utero* labeling of diverse LGE embryonic progenitor pools with functional studies of their neuronal offspring to define the relationship between progenitor diversity and the cellular and circuit diversity in the postnatal striatum. The labeling strategy distinguished two progenitor pools at a defined embryonic stage and allowed for tracking of their progeny into the postnatal striatum. Although this allowed us to identify neuronal offspring derived from different pools, it provided limited information on the lineage pathways taken by neurons. Therefore, how aIP and OP relate to other progenitors or follow different lineage routes (Pilz et al., 2013; Kelly et al., 2018) needs

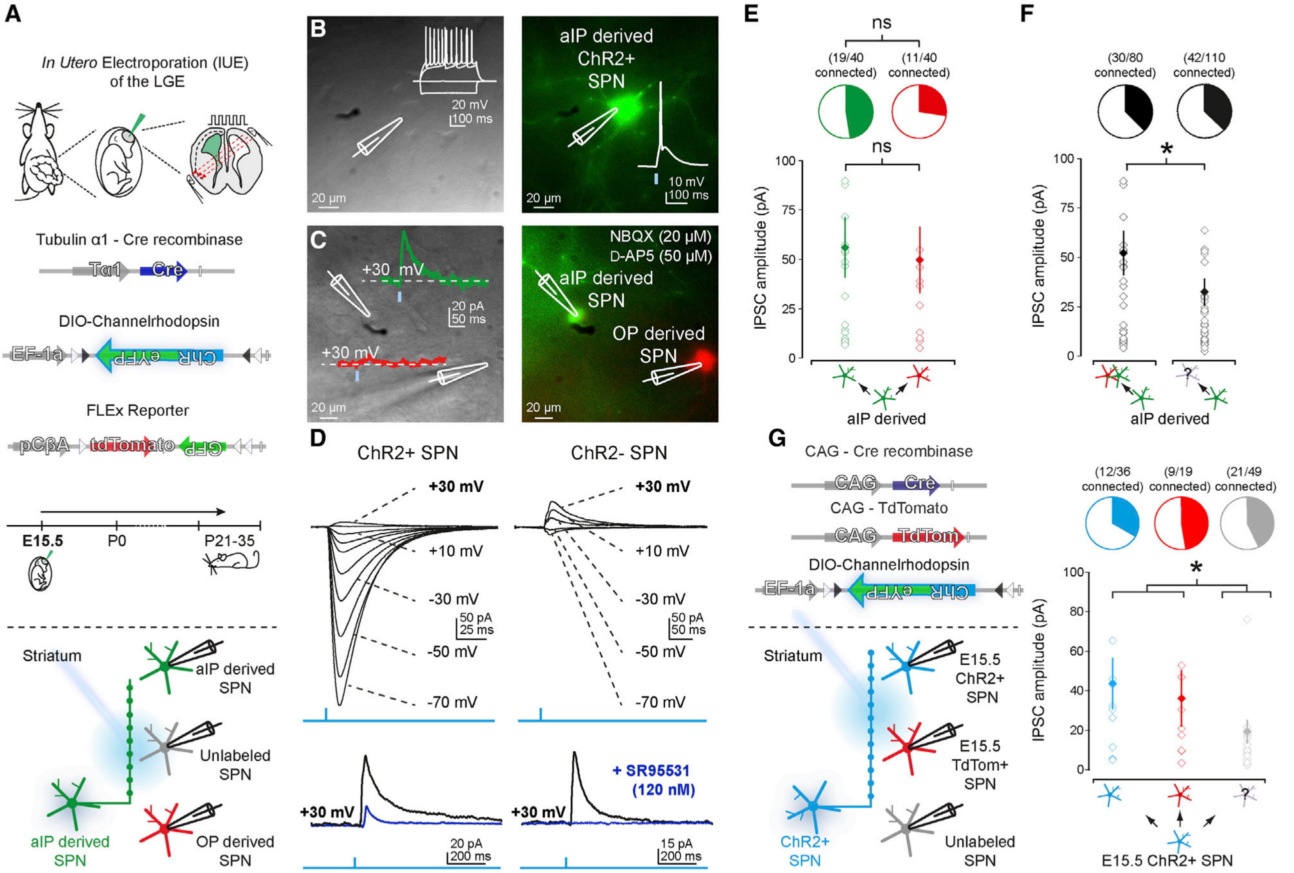

Figure 4. Birthdate rather than progenitor origin impacts local striatal inhibitory connection strength

(A) IUE of Tα1-Cre, FLEx, and DIO-ChR2-eYFP plasmids enabled the expression of ChR2-eYFP in aIP-derived SPNs and fluorescent labeling of aIP- and OP-derived SPNs. This allowed for optical activation of aIP-derived SPNs in combination with targeted whole-cell voltage-clamp recordings of progenitor-derived and unlabeled SPNs in postnatal tissue (P21–P35).

(B) Brief blue light pulses elicited single action potentials in aIP-derived ChR2$^+$ SPNs.

(C) Dual patch-clamp recording of aIP- and OP-derived SPNs. Inset: example traces of light evoked IPSCs in SPNs held at +30 mV.

(D) Detection of GABAergic currents in ChR2$^+$ and ChR2$^-$ SPNs was facilitated through voltage-clamp of SPNs at +30 mV and superfusion of 120 nM SR95531 allowing for subtraction of residual currents from baseline currents.

(E) Incidence and strength of inhibitory synaptic connections from aIP-derived SPNs to aIP- or OP-derived SPNs was similar.

(F) Optical activation of aIP-derived SPNs led to larger postsynaptic IPSCs in SPNs born from E15.5-labeled progenitors (left) compared to SPNs of unknown developmental origin (right).

(G) IUE of CAG-Cre, CAG-TdTom, and DIO-ChR2-eYFP plasmids allowed for expression of ChR2-eYFP and/or the fluorescent protein TdTom in E15.5-labeled SPNs. Photo-activation of ChR2$^+$ SPNs evoked larger postsynaptic IPSCs in SPNs labeled at E15.5 compared to SPNs of unknown developmental origin. Data are represented as mean ± SEM.

further study. Despite the heterogeneity of progenitor pools, both generated dSPNs and iSPNs with similar intrinsic electrical properties and morphologies and could be found throughout the postnatal striatum conforming to the general distribution of dSPNs and iSPNs (Gangarossa et al., 2013) and consistent with recent findings of active intermixing of young SPNs (Tinterri et al., 2018). The ability of distinct progenitor pools to generate both dSPNs and iSPNs has also been seen for other embryonic progenitor groups (Kelly et al., 2018), suggesting that additional factors beyond pool of origin contribute to SPN subtype generation (Tepper et al., 1998; Lobo et al., 2006; Franco et al., 2012; Kelly et al., 2018; Anderson et al., 2020; Sharma et al., 2020; Zhang et al., 2016; Lu et al., 2014).

We found that long-range excitatory glutamatergic synaptic inputs proved a particularly sensitive discriminator of aIP- and OP-derived SPNs. This suggests that diverse LGE progenitor pools can contribute to the establishment of parallel functional pathways through the basal ganglia (Alexander et al., 1986; Graybiel, 1990; Haber, 2008; Pan et al., 2010; Oh et al., 2014; Hintiryan et al., 2016; Hunnicutt et al., 2016; McGregor et al., 2019). Our data suggest that distinct information pathways through the striatum based on neural progenitor origin co-exists alongside integrated networks of converging multisensory pathways (Wilson et al., 1983; Nagy et al., 2006; Reig and Silberberg, 2014), divergent networks based on either dSPN/iSPN sub-divisions (Wall et al., 2013), and striosome-matrix sub-divisions

(McGregor et al., 2019; Matsushima and Graybiel, 2020). Lastly, our data provide further evidence for direct functional glutamatergic projections to DMS from VC areas (Hikosaka et al., 1989; Khibnik et al., 2014; Nagy et al., 2018) and mPFC (Choi et al., 2019; Loewke et al., 2020). Interestingly, our observations that mPFC strongly innervates aIP-derived SPNs, which preferentially innervate dSPNs, fits with the idea of fine-scale subnetworks dedicated to processing related information (Yoshimura and Callaway, 2005; Ko et al., 2011) because dSPNs are also preferentially driven by input from mPFC (Loewke et al., 2020). We suggest the generation of such subnetworks can be facilitated by diverse embryonic progenitor pools. Although we did not observe further changes in connection biases in mice up to 6 weeks old, whether or not these are retained over a lifetime remains to be studied.

We found that neurogenic stage, rather than progenitor origin (Ellender et al., 2019), determines the strength of local connections between SPNs, with SPNs of matching neurogenic stages forming stronger inhibitory connections, reminiscent of findings in hippocampus (Deguchi et al., 2011) and possibly acting alongside other factors (Goffin et al., 2010). It will be interesting to see whether stronger synaptic connections are also present among SPNs with matched birthdates from different embryonic periods. Together, our results suggest that SPNs, besides the canonical striatal types based on expression of specific and mutually exclusive markers such as D1- versus D2-type dopamine receptors or enrichment of MORs, can also be functionally defined by their embryonic progenitor origin and/or birthdate.

In conclusion, we show that the diversity in LGE embryonic progenitors contributes to striatal cellular diversity, particularly the establishment of parallel excitatory synaptic circuits into striatum. Whether these distinct and segregated synaptic circuits derived from different embryonic progenitor pools are maintained through the basal ganglia (Lee et al., 2020) and what their functional role is, whether embryonic progenitor diversity contributes to the phylogeny of striatal modules (Grillner and Robertson, 2016), how embryonic progenitor pools map on the SPN transcriptional heterogeneity (Gokce et al., 2016; Saunders et al., 2018; Zeisel et al., 2018; Märtin et al., 2019; Anderson et al., 2020), and whether embryonic progenitors are implicated in basal ganglia disorder etiology (Graybiel and Rauch, 2000; Shepherd, 2013; Tyler and Haydar, 2013; Gunaydin and Kreitzer, 2016) remains to be investigated.

STAR★METHODS

Detailed methods are provided in the online version of this paper and include the following:

- KEY RESOURCES TABLE
- RESOURCE AVAILABILITY
  - Lead contact
  - Materials availability
  - Data and code availability
- EXPERIMENTAL MODEL AND SUBJECT DETAILS
- METHOD DETAILS
  - Experimental design
  - In utero electroporation
  - Viral intracerebral injections
  - Slice preparation and recording conditions
  - Stimulation and recording protocols
  - Analysis of recordings
  - Histological analyses
  - Stereology and analysis of tissue
- QUANTIFICATION AND STATISTICAL ANALYSIS

SUPPLEMENTAL INFORMATION

Supplemental information can be found online at https://doi.org/10.1016/j.celrep.2021.109041.

ACKNOWLEDGMENTS

We would like to thank all Ellender lab members for advice and comments. We gratefully acknowledge Ulrich Müller and Tarik Haydar for providing reagents, Peter Magill and Colin Akerman for providing access to equipment, Ben Micklem for technical assistance, and Ricardo Marquez-Gomez, Monzilur Rahman, Rebecca Waterfield, Nicholas Pasternack, and Eoin Mac Reamoinn for help. The graphical abstract was created with BioRender. A.M.-D. was supported by an Imperial College research bursary and MRC studentship. J.G. was supported by a BBSRC studentship (BB/M011224/1). T.E. was supported by an MRC Career Development Award (MR/M009599/1).

AUTHOR CONTRIBUTIONS

F.v.H., A.M.-D., and T.E. designed the experiments. F.v.H., J.G., and T.E. performed the electrophysiology experiments and/or analysis. F.v.H., A.M.-D., J.G., R.K., and A.S. performed morphological and/or stereological analysis. All authors discussed the data. F.v.H., A.M.-D., and T.E. wrote the manuscript.

DECLARATION OF INTERESTS

The authors declare no conflict of interest.

Received: July 14, 2020
Revised: January 29, 2021
Accepted: April 6, 2021
Published: April 27, 2021

REFERENCES

Alexander, G.E., DeLong, M.R., and Strick, P.L. (1986). Parallel organization of functionally segregated circuits linking basal ganglia and cortex. Annu. Rev. Neurosci. *9*, 357–381.

Anderson, A.G., Kulkarni, A., Harper, M., and Konopka, G. (2020). Single-Cell Analysis of Foxp1-Driven Mechanisms Essential for Striatal Development. Cell Rep. *30*, 3051–3066.e7.

Arlotta, P., Molyneaux, B.J., Jabaudon, D., Yoshida, Y., and Macklis, J.D. (2008). Ctip2 controls the differentiation of medium spiny neurons and the establishment of the cellular architecture of the striatum. J. Neurosci. *28*, 622–632.

Arruda-Carvalho, M., Wu, W.C., Cummings, K.A., and Clem, R.L. (2017). Optogenetic Examination of Prefrontal-Amygdala Synaptic Development. J. Neurosci. *37*, 2976–2985.

Baumgart, J., and Baumgart, N. (2016). Cortex-, Hippocampus-, Thalamus-, Hypothalamus-, Lateral Septal Nucleus- and Striatum-specific In Utero Electroporation in the C57BL/6 Mouse. J. Vis. Exp. *107*, e53303.

Berndt, A., Schoenenberger, P., Mattis, J., Tye, K.M., Deisseroth, K., Hegemann, P., and Oertner, T.G. (2011). High-efficiency channelrhodopsins for fast neuronal stimulation at low light levels. Proc. Natl. Acad. Sci. USA *108*, 7595–7600.

Burke, D.A., Rotstein, H.G., and Alvarez, V.A. (2017). Striatal Local Circuitry: A New Framework for Lateral Inhibition. Neuron *96*, 267–284.

Cadwell, C.R., Scala, F., Fahey, P.G., Kobak, D., Mulherkar, S., Sinz, F.H., Papadopoulos, S., Tan, Z.H., Johnsson, P., Hartmanis, L., et al. (2020). Cell type composition and circuit organization of clonally related excitatory neurons in the juvenile mouse neocortex. eLife *9*, e52951.

Cepeda, C., Galvan, L., Holley, S.M., Rao, S.P., André, V.M., Botelho, E.P., Chen, J.Y., Watson, J.B., Deisseroth, K., and Levine, M.S. (2013). Multiple sources of striatal inhibition are differentially affected in Huntington's disease mouse models. J. Neurosci. *33*, 7393–7406.

Choi, K., Holly, E.N., Davatolhagh, M.F., Beier, K.T., and Fuccillo, M.V. (2019). Integrated anatomical and physiological mapping of striatal afferent projections. Eur. J. Neurosci. *49*, 623–636.

Chuhma, N., Tanaka, K.F., Hen, R., and Rayport, S. (2011). Functional connectome of the striatal medium spiny neuron. J. Neurosci. *31*, 1183–1192.

Cui, G., Jun, S.B., Jin, X., Pham, M.D., Vogel, S.S., Lovinger, D.M., and Costa, R.M. (2013). Concurrent activation of striatal direct and indirect pathways during action initiation. Nature *494*, 238–242.

Day, M., Wokosin, D., Plotkin, J.L., Tian, X., and Surmeier, D.J. (2008). Differential excitability and modulation of striatal medium spiny neuron dendrites. J. Neurosci. *28*, 11603–11614.

Deguchi, Y., Donato, F., Galimberti, I., Cabuy, E., and Caroni, P. (2011). Temporally matched subpopulations of selectively interconnected principal neurons in the hippocampus. Nat. Neurosci. *14*, 495–504.

Ellender, T.J., Huerta-Ocampo, I., Deisseroth, K., Capogna, M., and Bolam, J.P. (2011). Differential modulation of excitatory and inhibitory striatal synaptic transmission by histamine. J. Neurosci. *31*, 15340–15351.

Ellender, T.J., Avery, S.V., Mahfooz, K., von Klemperer, A., Nixon, S.L., Buchan, M.J., van Rheede, J.J., Gatti, A., Waites, C., Newey, S.E., and Akerman, C.J. (2018). Fine-scale excitatory cortical circuits reflect embryonic progenitor pools. bioRxiv. https://doi.org/10.1101/363069.

Ellender, T.J., Avery, S.V., Mahfooz, K., Scaber, J., von Klemperer, A., Nixon, S.L., Buchan, M.J., van Rheede, J.J., Gatti, A., Waites, C., et al. (2019). Embryonic progenitor pools generate diversity in fine-scale excitatory cortical subnetworks. Nat. Commun. *10*, 5224.

Flames, N., Pla, R., Gelman, D.M., Rubenstein, J.L., Puelles, L., and Marín, O. (2007). Delineation of multiple subpallial progenitor domains by the combinatorial expression of transcriptional codes. J. Neurosci. *27*, 9682–9695.

Franco, S.J., and Müller, U. (2013). Shaping our minds: stem and progenitor cell diversity in the mammalian neocortex. Neuron *77*, 19–34.

Franco, S.J., Gil-Sanz, C., Martinez-Garay, I., Espinosa, A., Harkins-Perry, S.R., Ramos, C., and Müller, U. (2012). Fate-restricted neural progenitors in the mammalian cerebral cortex. Science *337*, 746–749.

Franklin, K.B.J., and Paxinos, G. (2008). The Mouse Brain in Stereotaxic Coordinates (Elsevier Academic Press).

Gal, J.S., Morozov, Y.M., Ayoub, A.E., Chatterjee, M., Rakic, P., and Haydar, T.F. (2006). Molecular and morphological heterogeneity of neural precursors in the mouse neocortical proliferative zones. J. Neurosci. *26*, 1045–1056.

Gangarossa, G., Espallergues, J., Mailly, P., De Bundel, D., de Kerchove d'Exaerde, A., Hervé, D., Girault, J.A., Valjent, E., and Krieger, P. (2013). Spatial distribution of D1R- and D2R-expressing medium-sized spiny neurons differs along the rostro-caudal axis of the mouse dorsal striatum. Front. Neural Circuits *7*, 124.

Garas, F.N., Shah, R.S., Kormann, E., Doig, N.M., Vinciati, F., Nakamura, K.C., Dorst, M.C., Smith, Y., Magill, P.J., and Sharott, A. (2016). Secretagogin expression delineates functionally-specialized populations of striatal parvalbumin-containing interneurons. eLife *5*, e16088.

Garas, F.N., Kormann, E., Shah, R.S., Vinciati, F., Smith, Y., Magill, P.J., and Sharott, A. (2018). Structural and molecular heterogeneity of calretinin-expressing interneurons in the rodent and primate striatum. J. Comp. Neurol. *526*, 877–898.

Gerfen, C.R., Engber, T.M., Mahan, L.C., Susel, Z., Chase, T.N., Monsma, F.J., Jr., and Sibley, D.R. (1990). D1 and D2 dopamine receptor-regulated gene expression of striatonigral and striatopallidal neurons. Science *250*, 1429–1432.

Gertler, T.S., Chan, C.S., and Surmeier, D.J. (2008). Dichotomous anatomical properties of adult striatal medium spiny neurons. J. Neurosci. *28*, 10814–10824.

Goffin, D., Ali, A.B., Rampersaud, N., Harkavyi, A., Fuchs, C., Whitton, P.S., Nairn, A.C., and Jovanovic, J.N. (2010). Dopamine-dependent tuning of striatal inhibitory synaptogenesis. J. Neurosci. *30*, 2935–2950.

Gokce, O., Stanley, G.M., Treutlein, B., Neff, N.F., Camp, J.G., Malenka, R.C., Rothwell, P.E., Fuccillo, M.V., Südhof, T.C., and Quake, S.R. (2016). Cellular Taxonomy of the Mouse Striatum as Revealed by Single-Cell RNA-Seq. Cell Rep. *16*, 1126–1137.

Gong, S., Zheng, C., Doughty, M.L., Losos, K., Didkovsky, N., Schambra, U.B., Nowak, N.J., Joyner, A., Leblanc, G., Hatten, M.E., and Heintz, N. (2003). A gene expression atlas of the central nervous system based on bacterial artificial chromosomes. Nature *425*, 917–925.

Graybiel, A.M. (1990). Neurotransmitters and neuromodulators in the basal ganglia. Trends Neurosci. *13*, 244–254.

Graybiel, A.M., and Ragsdale, C.W., Jr. (1978). Histochemically distinct compartments in the striatum of human, monkeys, and cat demonstrated by acetylthiocholinesterase staining. Proc. Natl. Acad. Sci. USA *75*, 5723–5726.

Graybiel, A.M., and Rauch, S.L. (2000). Toward a neurobiology of obsessive-compulsive disorder. Neuron *28*, 343–347.

Graybiel, A.M., Aosaki, T., Flaherty, A.W., and Kimura, M. (1994). The basal ganglia and adaptive motor control. Science *265*, 1826–1831.

Grillner, S., and Robertson, B. (2016). The Basal Ganglia Over 500 Million Years. Curr. Biol. *26*, R1088–R1100.

Grillner, S., Hellgren, J., Ménard, A., Saitoh, K., and Wikström, M.A. (2005). Mechanisms for selection of basic motor programs–roles for the striatum and pallidum. Trends Neurosci. *28*, 364–370.

Gunaydin, L.A., and Kreitzer, A.C. (2016). Cortico-Basal Ganglia Circuit Function in Psychiatric Disease. Annu. Rev. Physiol. *78*, 327–350.

Guo, Q., Wang, D., He, X., Feng, Q., Lin, R., Xu, F., Fu, L., and Luo, M. (2015). Whole-brain mapping of inputs to projection neurons and cholinergic interneurons in the dorsal striatum. PLoS ONE *10*, e0123381.

Haber, S. (2008). Parallel and integrative processing through the Basal Ganglia reward circuit: lessons from addiction. Biol. Psychiatry *64*, 173–174.

Halliday, A.L., and Cepko, C.L. (1992). Generation and migration of cells in the developing striatum. Neuron *9*, 15–26.

Hammad, M., Schmidt, S.L., Zhang, X., Bray, R., Frohlich, F., and Ghashghaei, H.T. (2015). Transplantation of GABAergic Interneurons into the Neonatal Primary Visual Cortex Reduces Absence Seizures in Stargazer Mice. Cereb. Cortex *25*, 2970–2979.

Hikosaka, O., Sakamoto, M., and Usui, S. (1989). Functional properties of monkey caudate neurons. II. Visual and auditory responses. J. Neurophysiol. *61*, 799–813.

Hintiryan, H., Foster, N.N., Bowman, I., Bay, M., Song, M.Y., Gou, L., Yamashita, S., Bienkowski, M.S., Zingg, B., Zhu, M., et al. (2016). The mouse cortico-striatal projectome. Nat. Neurosci. *19*, 1100–1114.

Hunnicutt, B.J., Jongbloets, B.C., Birdsong, W.T., Gertz, K.J., Zhong, H., and Mao, T. (2016). A comprehensive excitatory input map of the striatum reveals novel functional organization. eLife *5*, e19103.

Johansson, Y., and Silberberg, G. (2020). The Functional Organization of Cortical and Thalamic Inputs onto Five Types of Striatal Neurons Is Determined by Source and Target Cell Identities. Cell Rep. *30*, 1178–1194.e3.

Kelly, S.M., Raudales, R., He, M., Lee, J.H., Kim, Y., Gibb, L.G., Wu, P., Matho, K., Osten, P., Graybiel, A.M., and Huang, Z.J. (2018). Radial Glial Lineage Progression and Differential Intermediate Progenitor Amplification Underlie Striatal Compartments and Circuit Organization. Neuron *99*, 345–361.e4.

Khibnik, L.A., Tritsch, N.X., and Sabatini, B.L. (2014). A direct projection from mouse primary visual cortex to dorsomedial striatum. PLoS ONE *9*, e104501.

Ko, H., Hofer, S.B., Pichler, B., Buchanan, K.A., Sjöström, P.J., and Mrsic-Flogel, T.D. (2011). Functional specificity of local synaptic connections in neocortical networks. Nature *473*, 87–91.

Kowalczyk, T., Pontious, A., Englund, C., Daza, R.A., Bedogni, F., Hodge, R., Attardo, A., Bell, C., Huttner, W.B., and Hevner, R.F. (2009). Intermediate neuronal progenitors (basal progenitors) produce pyramidal-projection neurons for all layers of cerebral cortex. Cereb. Cortex *19*, 2439–2450.

Krajeski, R.N., Macey-Dare, A., van Heusden, F., Ebrahimjee, F., and Ellender, T.J. (2019). Dynamic postnatal development of the cellular and circuit properties of striatal D1 and D2 spiny projection neurons. J. Physiol. *597*, 5265–5293.

Kravitz, A.V., Freeze, B.S., Parker, P.R., Kay, K., Thwin, M.T., Deisseroth, K., and Kreitzer, A.C. (2010). Regulation of parkinsonian motor behaviours by optogenetic control of basal ganglia circuitry. Nature *466*, 622–626.

Laubach, M., Amarante, L.M., Swanson, K., and White, S.R. (2018). What, If Anything, Is Rodent Prefrontal Cortex? eNeuro *5*, ENEURO.0315-18.2018.

Lee, T., Kaneko, T., Taki, K., and Mizuno, N. (1997). Preprodynorphin-, preproenkephalin-, and preprotachykinin-expressing neurons in the rat neostriatum: an analysis by immunocytochemistry and retrograde tracing. J. Comp. Neurol. *386*, 229–244.

Lee, J., Wang, W., and Sabatini, B.L. (2020). Anatomically segregated basal ganglia pathways allow parallel behavioral modulation. Nat. Neurosci. *23*, 1388–1398.

Lobo, M.K., Karsten, S.L., Gray, M., Geschwind, D.H., and Yang, X.W. (2006). FACS-array profiling of striatal projection neuron subtypes in juvenile and adult mouse brains. Nat. Neurosci. *9*, 443–452.

Loewke, A.C., Minerva, A.R., Nelson, A.B., Kreitzer, A.C., and Gunaydin, L.A. (2020). Fronto-striatal projections regulate approach-avoidance conflict. bioRxiv. https://doi.org/10.1101/2020.03.05.979708.

Lu, J.Y., Cheng, Y.F., Wang, X.H., Woodson, K., Kemper, C., Disney, E., and Wang, J. (2019). Alcohol intake enhances glutamatergic transmission from D2 receptor-expressing afferents onto D1 receptor-expressing medium spiny neurons in the dorsomedial striatum. Neuropsychopharmacology *44*, 1123–1131.

Lu, K.M., Evans, S.M., Hirano, S., and Liu, F.C. (2014). Dual role for Islet-1 in promoting striatonigral and repressing striatopallidal genetic programs to specify striatonigral cell identity. Proc. Natl. Acad. Sci. USA *111*, E168–E177.

Märtin, A., Calvigioni, D., Tzortzi, O., Fuzik, J., Wärnberg, E., and Meletis, K. (2019). A Spatiomolecular Map of the Striatum. Cell Rep. *29*, 4320–4333.e5.

Mason, H.A., Rakowiecki, S.M., Raftopoulou, M., Nery, S., Huang, Y., Gridley, T., and Fishell, G. (2005). Notch signaling coordinates the patterning of striatal compartments. Development *132*, 4247–4258.

Matsuda, T., and Cepko, C.L. (2007). Controlled expression of transgenes introduced by in vivo electroporation. Proc. Natl. Acad. Sci. USA *104*, 1027–1032.

Matsushima, A., and Graybiel, A.M. (2020). Combinatorial Developmental Controls on Striatonigral Circuits. Cell Rep. *31*, 107778.

McGregor, M.M., McKinsey, G.L., Girasole, A.E., Bair-Marshall, C.J., Rubenstein, J.L.R., and Nelson, A.B. (2019). Functionally Distinct Connectivity of Developmentally Targeted Striosome Neurons. Cell Rep. *29*, 1419–1428.e5.

Nagel, G., Szellas, T., Huhn, W., Kateriya, S., Adeishvili, N., Berthold, P., Ollig, D., Hegemann, P., and Bamberg, E. (2003). Channelrhodopsin-2, a directly light-gated cation-selective membrane channel. Proc. Natl. Acad. Sci. USA *100*, 13940–13945.

Nagy, A., Eördegh, G., Paróczy, Z., Márkus, Z., and Benedek, G. (2006). Multisensory integration in the basal ganglia. Eur. J. Neurosci. *24*, 917–924.

Nagy, A.J., Takeuchi, Y., and Berényi, A. (2018). Coding of self-motion-induced and self-independent visual motion in the rat dorsomedial striatum. PLoS Biol. *16*, e2004712.

Noctor, S.C., Flint, A.C., Weissman, T.A., Dammerman, R.S., and Kriegstein, A.R. (2001). Neurons derived from radial glial cells establish radial units in neocortex. Nature *409*, 714–720.

Noctor, S.C., Martínez-Cerdeño, V., Ivic, L., and Kriegstein, A.R. (2004). Cortical neurons arise in symmetric and asymmetric division zones and migrate through specific phases. Nat. Neurosci. *7*, 136–144.

Oh, S.W., Harris, J.A., Ng, L., Winslow, B., Cain, N., Mihalas, S., Wang, Q., Lau, C., Kuan, L., Henry, A.M., et al. (2014). A mesoscale connectome of the mouse brain. Nature *508*, 207–214.

Olsson, M., Björklund, A., and Campbell, K. (1998). Early specification of striatal projection neurons and interneuronal subtypes in the lateral and medial ganglionic eminence. Neuroscience *84*, 867–876.

Pan, W.X., Mao, T., and Dudman, J.T. (2010). Inputs to the dorsal striatum of the mouse reflect the parallel circuit architecture of the forebrain. Front. Neuroanat. *4*, 147.

Pilz, G.A., Shitamukai, A., Reillo, I., Pacary, E., Schwausch, J., Stahl, R., Ninkovic, J., Snippert, H.J., Clevers, H., Godinho, L., et al. (2013). Amplification of progenitors in the mammalian telencephalon includes a new radial glial cell type. Nat. Commun. *4*, 2125.

Planert, H., Szydlowski, S.N., Hjorth, J.J., Grillner, S., and Silberberg, G. (2010). Dynamics of synaptic transmission between fast-spiking interneurons and striatal projection neurons of the direct and indirect pathways. J. Neurosci. *30*, 3499–3507.

Reid, C.B., and Walsh, C.A. (2002). Evidence of common progenitors and patterns of dispersion in rat striatum and cerebral cortex. J. Neurosci. *22*, 4002–4014.

Reig, R., and Silberberg, G. (2014). Multisensory integration in the mouse striatum. Neuron *83*, 1200–1212.

Saunders, A., Johnson, C.A., and Sabatini, B.L. (2012). Novel recombinant adeno-associated viruses for Cre activated and inactivated transgene expression in neurons. Front. Neural Circuits *6*, 47.

Saunders, A., Macosko, E.Z., Wysoker, A., Goldman, M., Krienen, F.M., de Rivera, H., Bien, E., Baum, M., Bortolin, L., Wang, S., et al. (2018). Molecular Diversity and Specializations among the Cells of the Adult Mouse Brain. Cell *174*, 1015–1030.e16.

Schambra, U.B., and Schambra, U.B.A.b. (2008). Prenatal Mouse Brain Atlas (Springer).

Sharma, N., Flaherty, K., Lezgiyeva, K., Wagner, D.E., Klein, A.M., and Ginty, D.D. (2020). The emergence of transcriptional identity in somatosensory neurons. Nature *577*, 392–398.

Sharott, A., Vinciati, F., Nakamura, K.C., and Magill, P.J. (2017). A Population of Indirect Pathway Striatal Projection Neurons Is Selectively Entrained to Parkinsonian Beta Oscillations. J. Neurosci. *37*, 9977–9998.

Shepherd, G.M. (2013). Corticostriatal connectivity and its role in disease. Nat. Rev. Neurosci. *14*, 278–291.

Shitamukai, A., Konno, D., and Matsuzaki, F. (2011). Oblique radial glial divisions in the developing mouse neocortex induce self-renewing progenitors outside the germinal zone that resemble primate outer subventricular zone progenitors. J. Neurosci. *31*, 3683–3695.

Stancik, E.K., Navarro-Quiroga, I., Sellke, R., and Haydar, T.F. (2010). Heterogeneity in ventricular zone neural precursors contributes to neuronal fate diversity in the postnatal neocortex. J. Neurosci. *30*, 7028–7036.

Stell, B.M., and Mody, I. (2002). Receptors with different affinities mediate phasic and tonic GABA(A) conductances in hippocampal neurons. J. Neurosci. *22*, RC223.

Stenman, J., Toresson, H., and Campbell, K. (2003). Identification of two distinct progenitor populations in the lateral ganglionic eminence: implications for striatal and olfactory bulb neurogenesis. J. Neurosci. *23*, 167–174.

Tan, S.S., and Breen, S. (1993). Radial mosaicism and tangential cell dispersion both contribute to mouse neocortical development. Nature *362*, 638–640.

Taverna, S., Ilijic, E., and Surmeier, D.J. (2008). Recurrent collateral connections of striatal medium spiny neurons are disrupted in models of Parkinson's disease. J. Neurosci. *28*, 5504–5512.

Taverna, E., Götz, M., and Huttner, W.B. (2014). The cell biology of neurogenesis: toward an understanding of the development and evolution of the neocortex. Annu. Rev. Cell Dev. Biol. *30*, 465–502.

Tecuapetla, F., Jin, X., Lima, S.Q., and Costa, R.M. (2016). Complementary Contributions of Striatal Projection Pathways to Action Initiation and Execution. Cell *166*, 703–715.

Tepper, J.M., Sharpe, N.A., Koós, T.Z., and Trent, F. (1998). Postnatal development of the rat neostriatum: electrophysiological, light- and electron-microscopic studies. Dev. Neurosci. *20*, 125–145.

Tinterri, A., Menardy, F., Diana, M.A., Lokmane, L., Keita, M., Coulpier, F., Lemoine, S., Mailhes, C., Mathieu, B., Merchan-Sala, P., et al. (2018). Active intermixing of indirect and direct neurons builds the striatal mosaic. Nat. Commun. *9*, 4725.

Tucker, E.S., Segall, S., Gopalakrishna, D., Wu, Y., Vernon, M., Polleux, F., and Lamantia, A.S. (2008). Molecular specification and patterning of progenitor cells in the lateral and medial ganglionic eminences. J. Neurosci. *28*, 9504–9518.

Tyler, W.A., and Haydar, T.F. (2013). Multiplex genetic fate mapping reveals a novel route of neocortical neurogenesis, which is altered in the Ts65Dn mouse model of Down syndrome. J. Neurosci. *33*, 5106–5119.

Tyler, W.A., Medalla, M., Guillamon-Vivancos, T., Luebke, J.I., and Haydar, T.F. (2015). Neural precursor lineages specify distinct neocortical pyramidal neuron types. J. Neurosci. *35*, 6142–6152.

van der Kooy, D., and Fishell, G. (1987). Neuronal birthdate underlies the development of striatal compartments. Brain Res. *401*, 155–161.

Wall, N.R., De La Parra, M., Callaway, E.M., and Kreitzer, A.C. (2013). Differential innervation of direct- and indirect-pathway striatal projection neurons. Neuron *79*, 347–360.

Wamsley, B., and Fishell, G. (2017). Genetic and activity-dependent mechanisms underlying interneuron diversity. Nat. Rev. Neurosci. *18*, 299–309.

Wang, X., Tsai, J.W., LaMonica, B., and Kriegstein, A.R. (2011). A new subtype of progenitor cell in the mouse embryonic neocortex. Nat. Neurosci. *14*, 555–561.

Wilson, J.S., Hull, C.D., and Buchwald, N.A. (1983). Intracellular studies of the convergence of sensory input on caudate neurons of cat. Brain Res. *270*, 197–208.

Wonders, C.P., and Anderson, S.A. (2006). The origin and specification of cortical interneurons. Nat. Rev. Neurosci. *7*, 687–696.

Xu, Z., Liang, Q., Song, X., Zhang, Z., Lindtner, S., Li, Z., Wen, Y., Liu, G., Guo, T., Qi, D., et al. (2018). SP8 and SP9 coordinately promote D2-type medium spiny neuron production by activating *Six3* expression. Development *145*, dev165456.

Yin, H.H., and Knowlton, B.J. (2006). The role of the basal ganglia in habit formation. Nat. Rev. Neurosci. *7*, 464–476.

Yoshimura, Y., and Callaway, E.M. (2005). Fine-scale specificity of cortical networks depends on inhibitory cell type and connectivity. Nat. Neurosci. *8*, 1552–1559.

Yu, Y.C., Bultje, R.S., Wang, X., and Shi, S.H. (2009). Specific synapses develop preferentially among sister excitatory neurons in the neocortex. Nature *458*, 501–504.

Yu, Y.C., He, S., Chen, S., Fu, Y., Brown, K.N., Yao, X.H., Ma, J., Gao, K.P., Sosinsky, G.E., Huang, K., and Shi, S.H. (2012). Preferential electrical coupling regulates neocortical lineage-dependent microcircuit assembly. Nature *486*, 113–117.

Zeisel, A., Hochgerner, H., Lönnerberg, P., Johnsson, A., Memic, F., van der Zwan, J., Häring, M., Braun, E., Borm, L.E., La Manno, G., et al. (2018). Molecular Architecture of the Mouse Nervous System. Cell *174*, 999–1014.e22.

Zhang, F., Gradinaru, V., Adamantidis, A.R., Durand, R., Airan, R.D., de Lecea, L., and Deisseroth, K. (2010). Optogenetic interrogation of neural circuits: technology for probing mammalian brain structures. Nat. Protoc. *5*, 439–456.

Zhang, Q., Zhang, Y., Wang, C., Xu, Z., Liang, Q., An, L., Li, J., Liu, Z., You, Y., He, M., et al. (2016). The Zinc Finger Transcription Factor Sp9 Is Required for the Development of Striatopallidal Projection Neurons. Cell Rep. *16*, 1431–1444.

CellPress
OPEN ACCESS

STAR★METHODS

KEY RESOURCES TABLE

REAGENT or RESOURCE	SOURCE	IDENTIFIER
Antibodies		
Streptavidin-AlexaFluor405	ThermoFisher Scientific	Cat#:S32351
Rat anti-CTIP2	Abcam	Cat#:ab14865; RRID:AB_2064130
Rabbit anti-pre-proenkephalin	LifeSpan Biosciences	Cat#:LS-C23084; RRID:AB_902714
Goat anti-rat AlexaFluor647	ThermoFisher Scientific	Cat#:A-21247; RRID:AB_141778
Goat anti-rabbit AlexaFluor555	ThermoFisher Scientific	Cat#:A-21429; RRID:AB_2535850
Goat anti-rabbit AlexaFluor488	ThermoFisher Scientific	Cat#:A32731; RRID:AB_2866491
Chicken anti-GFP	Aves Labs	Cat# GFP-1020; RRID:AB_10000240
Rat anti-RFP	Chromotek	Cat# 5f8-100; RRID:AB_2336064
Goat anti-chicken AlexaFluor488	Life Technologies	Cat#:A11039; RRID:AB_142924
Goat anti-rat AlexaFluor555	Life Technologies	Cat#:A-21434; RRID:AB_141733
Rabbit anti-pH3	Millipore	Cat#:06-570; RRID:AB_310177
Goat anti-MOR	Immunostar	Cat#:24216; RRID:AB_572251
Donkey anti-rabbit AlexaFluor647	Life Technologies	Cat#:A31573; RRID:AB_2536183
Goat anti-parvalbumin	Synaptic Systems	Cat#:; 195004; RRID:AB_2156476
Rabbit anti-NPY	ImmunoStar	Cat#:; 22940; RRID:AB_2307354
Rabbit anti-calretinin	Synaptic Systems	Cat#:; 214102; RRID:AB_2228331
Mouse anti-DARPP32	BD Bioscience	Cat#:; 611520; RRID:AB_398980
Donkey anti-guinea pig AlexaFluor594	Life Technologies	Cat#:; A21450; RRID:AB_2534069
Donkey anti-mouse AlexaFluor488	Life Technologies	Cat#:; A11001; RRID:AB_398980
Chemicals, peptides, and recombinant proteins		
Biocytin	Sigma	Cat#B1758; CAS:98930-70-2
SR95531	Tocris	Cat#1262; CAS:104104-50-9
CGP52432	Tocris	Cat#1246; CAS:139667-74-6
NBQX	Tocris	Cat#1044; CAS:118876-58-7
D-AP5	Tocris	Cat#0106; CAS:79055-68-8
DAPI (4',6-Diaminidino-2-phenylindole, Dihydrochloride)	ThermoFisher Scientific	Cat#D1306; RRID:AB_2629482
DAB (3,3′-Diaminobenzidine)	Sigma	Cat#D4168
VECTASTAIN Elite ABC-Peroxidase Kit, Vector Laboratories	Vector Laboratories	Cat#PK-6100; RRID:AB_2336819
Vectashield Antifade Mounting Medium	Vector Laboratories	Cat#H-1000; RRID:AB_2336789
NDS (Normal donkey serum)	Vector Laboratories	Cat#017-000-121; RRID:AB_2337258
NGS (Normal goat serum)	Vector Laboratories	Cat#S-1000; RRID:AB_2336615
Experimental models: viral strains		
AAV1-CaMKIIa-hChR2(H134R)-EYFP	Addgene	#26969-AAV1; RRID:Addgene_26969
Experimental models		
Mouse: C57BL/6	Charles River	RRID:IMSR_CRL:027
Mouse: D1-GFP	MMRRC	RRID:MMRRC_000297-MU
Mouse: D2-GFP	MMRRC	RRID:MMRRC_000230-UNC
Deposited data		
Neuronal reconstructions	This manuscript	https://Neuromorpho.org

(*Continued on next page*)

Continued		
REAGENT or RESOURCE	SOURCE	IDENTIFIER
Software and algorithms		
WinWCP software	University of Strathclyde	http://spider.science.strath.ac.uk; RRID:SCR_014713
Igor Pro	Wavemetrics	https://www.wavemetrics.com/; RRID:SCR_000325
ZEN	Zeiss	RRID:SCR_013672
Open Lab	Perkin Elmer	RRID:SCR_012158
ImageJ	N/A	RRID:SCR_003070
StereoInvestigator	MBF Biosciences	RRID:SCR_002526
Neurolucida	MBF Biosciences	RRID:SCR_016788
Neuroexplorer	MBF Biosciences	RRID:SCR_001775
SPSS 17.0	IBM SPSS Statistics	RRID:SCR_002865
Graphpad Prism 5.0	Graphpad Software	RRID:SCR_002798
Recombinant DNA		
Tα1-Cre	Stancik et al., 2010	N/A
CβA-FLEx	Franco et al., 2012	N/A
pAAV-EF1a-doublefloxed-hChR2(H134R)-mCherry-WPRE-HGHpA	Saunders et al., 2012	Cat#20297; RRID:Addgene_20297
pAAV-EF1a-doublefloxed-hChR2(H134R)-eYFP-WPRE- HGHpA	Zhang et al., 2010	Cat#20298; RRID:Addgene_20298
CAG-Cre	Matsuda and Cepko, 2007	Cat#13775; RRID:Addgene_13775
CAG-TdTomato	N/A	Cat##59462; RRID:Addgene_59462
Other		
BTX ECM 830 pulse generator	Genetronics	RRID:SCR_016841
CUY650P3 Tweezertrode	Sonidel	N/A
HM650V microtome	Microm	N/A
CoolLED pE-300 system	CoolLED	N/A
LSM710 microscope	Zeiss	RRID:SCR_018063
DM5000B microscope	Leica	RRID:SCR_012158
Zeiss Imager M2 microscope	Zeiss	RRID:SCR_018876
VT1000S microtome	Leica Microsystems	RRID:SCR_016495

RESOURCE AVAILABILITY

Lead contact

Further information and requests for resources and reagents should be directed to and will be fulfilled by the Lead Contact, Tommas Ellender (tommas.ellender@pharm.ox.ac.uk).

Materials availability

This study did not generate new unique reagents.

Data and code availability

Original SPN reconstruction data have been deposited on Neuromorpho: https://Neuromorpho.org.

EXPERIMENTAL MODEL AND SUBJECT DETAILS

All experiments were carried out on C57BL/6 wild-type and heterozygous D1-GFP or D2-GFP mice of both sexes with *ad libitum* access to food and water. The D1-GFP or D2-GFP BAC transgenic mice report subtypes of the dopamine receptor, either D1 or D2, by the presence of GFP (Mutant Mouse Regional Resource Centers, MMRRC) and correspond respectively to the D1-expressing direct pathway (dSPNs) and D2-expressing indirect pathway (iSPNs). Details of the mice and the methods of BAC mice production have been published (Gong et al., 2003) and can be found on the GENSAT website [GENSAT (2009) The Gene Expression Nervous System Atlas (GENSAT)

CellPress
OPEN ACCESS

Project. In: NINDS, Contracts N01NS02331 and HHSN271200723701C, The Rockefeller University (New York), http://www.gensat.org/index.html]. The BAC transgenic mice were backcrossed to a C57BL/6 background over 20+ generations prior to use and kept as a heterozygous mouse line. All mice were group housed, bred, IVC housed in a temperature-controlled animal facility (normal 12:12 h light/dark cycles) and used in accordance with the UK Animals (Scientific Procedures) Act (1986) and with prior approval from the local Ethical Review committees. Females were checked for plugs daily; the day of the plug was considered embryonic day (E)0.5.

METHOD DETAILS

Experimental design

This study did not involve randomization or blinding, except for morphological reconstructions, which were blind to neuron type, and study of long-range inputs to SPNs, which were blind to cortical injection site. We did not estimate sample-size before carrying out the study. No data or subjects were excluded from the analysis.

In utero electroporation

In utero electroporation (IUE) was performed using standard procedures. In short, pregnant females were anaesthetized using isoflurane and their uterine horns were exposed by midline laparotomy. A mixture of plasmid DNA and 0.03% fast green dye was injected intraventricularly using pulled micropipettes through the uterine wall and amniotic sac. Plasmid DNA included: (i) 'Tα1-Cre', in which the gene for Cre recombinase is under the control of a portion of the Tα1 promoter (Stancik et al., 2010); (ii) 'CβA-FLEx' which uses the chicken β-*actin* promoter to control a flexible excision (FLEx) cassette in which Cre recombination switches expression from TdTomato fluorescent protein to enhanced green fluorescent protein (Franco et al., 2012); and (iii) 'DIO-ChR2-mCherry' (pAAV-EF1a-doublefloxed-hChR2(H134R)-mCherry-WPRE-HGHpA; Addgene #20297), in which Cre recombination turns on the expression of channelrhodopsin-2 (ChR2) under the control of the human elongation factor-1a promoter (Saunders et al., 2012), (iv) 'DIO-ChR2-eYFP' (pAAV-EF1a-doublefloxed-hChR2(H134R)-eYFP-WPRE- HGHpA; Addgene #20298), in which Cre recombination turns on the expression of ChR2 under the control of the human elongation factor-1a promoter (Zhang et al., 2010); (v) 'CAG-Cre', in which the gene for Cre recombinase is under the control the CAG promoter (Matsuda and Cepko, 2007) or (vi) 'CAG-TdTomato', in which the expression of the TdTomato fluorescent protein is under the control of the CAG promoter. Total volume injected per pup was ~1 μl. Tα1-Cre and CβA-FLEx constructs (and other combinations of constructs) were thoroughly mixed and injected at equal ratios (stock concentration of all plasmids was ~3.0 μg/μl, so final concentration of two plasmid constructs in 1:1 ratio was 1.5 μg/μl) and exhibited robust and faithful expression at these concentrations (Ellender et al., 2019). The negative pole of the Tweezertrode (Sonidel) was placed just above the primordial ear outside the uterine muscle and the positive pole was placed slightly lower at the contralateral cheek region (Baumgart and Baumgart, 2016). Five pulses (50 ms duration separated by 200 - 950 ms) at 42-55V were given with a BTX ECM 830 pulse generator (Genetronics). Typically, around 80% of the pups underwent electroporation. Afterward the uterine horns were placed back inside the abdomen, the cavity was filled with warm physiological saline and the abdominal muscle and skin incisions were closed with vicryl and prolene sutures, respectively. Dams were placed back in a clean cage and monitored closely until the birth of the pups.

Viral intracerebral injections

Postnatal day 1-3 pups were anaesthetized by hypothermia and small volume injections (150 nl) of AAV1-CAMKII-hChR2(H134R)-EYF (Addgene, #26969-AAV1) were unilaterally made in medial prefrontal cortex (mPFC) or visual cortex (VC) of electroporated pups while the mice were stabilized on ice. Injections were ipsilateral to electroporated striatal regions, with approximate coordinates for mPFC (from Bregma, AP: +0.3-0.5, ML: 0.1, DV: −0.5-0.9) (Arruda-Carvalho et al., 2017) and for VC (from Lamda, AP: +0.1-0.3, ML: 1.5-2.5, DV: −0.7-1.0) (Hammad et al., 2015).

Slice preparation and recording conditions

Acute striatal slices were made from postnatal animals at 3-5 weeks of age, corresponding to young adulthood (Krajeski et al., 2019), unless otherwise indicated. Animals were anaesthetized with isoflurane and then decapitated. Coronal slices of 350-400 μm were cut using a vibrating microtome (Microm HM650V). Slices were prepared in artificial cerebrospinal fluid (aCSF) containing (in mM): 65 Sucrose, 85 NaCl, 2.5 KCl, 1.25 NaH_2PO_4, 7 $MgCl_2$, 0.5 $CaCl_2$, 25 $NaHCO_3$ and 10 glucose, pH 7.2-7.4, bubbled with carbogen gas (95% O_2 / 5% CO_2). Slices were immediately transferred to a storage chamber of recording aCSF containing (in mM): 130 NaCl, 3.5 KCl, 1.2 NaH_2PO_4, 2 $MgCl_2$, 2 $CaCl_2$, 24 $NaHCO_3$ and 10 glucose, pH 7.2 - 7.4, at 32°C and bubbled with carbogen gas until used for recording. Striatal slices were transferred to a recording chamber and continuously superfused with aCSF bubbled with carbogen gas with the same composition as the storage solution (32°C and perfusion speed of 2 ml/min). Whole-cell patch-clamp recordings were performed from one to four SPNs simultaneously in dorsal striatum using glass pipettes (~6MΩ), pulled from standard wall borosilicate glass capillaries and contained for whole-cell current-clamp (in mM): 110 potassium gluconate, 40 HEPES, 2 ATP-Mg, 0.3 Na-GTP, 4 NaCl and 4 mg/ml biocytin (pH 7.2-7.3; osmolarity, 290-300 mosmol/l) and for whole-cell voltage-clamp (in mM): 120 cesium gluconate, 40 HEPES, 4 NaCl, 2 ATP-Mg, 0.3 Na-GTP, 0.2 QX-314 and 4 mg/ml biocytin (pH 7.2–7.3; osmolarity, 290-300 mosmol/L). Recordings were made using Multiclamp 700B amplifiers and filtered at 4kHz and acquired using an InstruTECH ITC-18 analog/digital board and WinWCP software (University of Strathclyde) at 10 kHz.

Stimulation and recording protocols

Hyperpolarizing and depolarizing current steps (−500pA to +500pA) were used to assess the intrinsic properties of recorded SPNs in the dorsomedial striatum including input resistance and spike threshold (using small incremental current steps), as well as the properties of action potentials (amplitude, frequency and duration). Properties were assessed immediately on break-in. A distinction between striatal SPNs and interneurons was made based on our previous experience (Ellender et al., 2011; Krajeski et al., 2019) and involved a combined assessment of resting membrane potential, input resistance, delay to firing and overall action potential frequency, and if in doubt neurons were not taken further for experiments. Activation of excitatory cortical afferents was performed in the presence of blockers of inhibitory GABAergic transmission including the $GABA_A$-receptor antagonist SR95531 (1 μM) and the $GABA_B$-receptor antagonist CGP52432 (2 μM). Activation of inhibitory local connections between SPNs was performed in the presence of blockers of excitatory glutamatergic transmission including the AMPA/kainate-receptor antagonist NBQX (20 μM) and the NMDA-receptor antagonist D-AP5 (50 μM). In these sets of experiments all SPNs were voltage-clamped at +30mV and optical activation led to inhibitory postsynaptic currents and/or photocurrents in recorded SPNs. To distinguish between postsynaptic currents and photocurrents slices were superfused with 120 nM SR95531 for more than 8 minutes, while keeping SPNs at −70mV, after which SPNs were again voltage-clamped at +30mV. Photoactivation of ChR2 was achieved using widefield 2-5 ms duration light pulses of ~1 mW via a TTL triggered CoolLED pE-300 system (CoolLED, Andover, UK). Afferents were activated every 5-10 s with up to 20 repetitions and excitatory postsynaptic responses (EPSPs) or inhibitory postsynaptic responses (IPSCs) recorded from the patched SPNs. Optogenetic circuit mapping experiments consisted of single up to quadruple simultaneous patch-clamp recordings of different SPNs in dorsomedial striatal regions containing strong $ChR2^+$ neuronal or axonal labeling. In the case of single neuron recordings they were performed sequentially with the same region. SPNs were sampled within 50 – 100 μm of each other and stimulation parameters were kept constant across recording days.

Analysis of recordings

Data were analyzed offline using custom written programs in Igor Pro (Wavemetrics). The input resistance was calculated from the observed membrane potential change after hyperpolarizing the membrane potential with a set current injection. The membrane time constant was taken as the time it takes for a change in potential to reach 63% of its final value. The spike threshold was the membrane voltage at which the SPN generated an action potential. The action potential amplitude was taken from the peak amplitude of the individual action potentials relative to the average steady-state membrane depolarization during positive current injection. Action potential duration was taken as the duration between the upward and downward stroke of the action potential at 25% of the peak amplitude. Optically evoked excitatory postsynaptic potentials (EPSPs) and inhibitory postsynaptic current (IPSCs) were defined as upward or downward deflections of more than 2 standard deviations (SD) from baseline as measured from averaged synaptic responses generated after filtering and averaging original traces (0.1 Hz high-pass filter and 500 Hz low-pass filter) and used for analysis of synaptic properties. To isolate the IPSC component from combined photo- and synaptic currents in ChR2 expression neurons the residual current after superfusion of SR95531 (120 nM) were subtracted from the combined current. Synaptic properties include measurements of peak amplitude, duration (measured from the start of the upward/downward stroke of the event until its return to the pre-event baseline), rise time (time between 10% and 90% of the peak amplitude) and decay time (measured as the time from peak amplitude until the event returned to 50% of peak amplitude).

Histological analyses

Following whole-cell patch-clamp recordings the brain slices were fixed in 4% paraformaldehyde in 0.1 M phosphate buffer (PB; pH 7.4). Biocytin-filled neurons were visualized by incubating sections in 1:10,000 streptavidin AlexaFluor405-conjugated antibodies (ThermoFisher Scientific). Visualized neurons were labeled for COUP TF1-interacting protein 2 (CTIP2, 1:1000) and pre-proenkephalin (PPE, 1:1000) in PBS containing 0.3% Triton X-100 (PBS-Tx) overnight at 4°C, followed by incubation with goat-anti-rat AlexaFluor647 (1:500) and goat-anti-rabbit AlexaFluor555 (1:500) or goat-anti-rabbit AlexaFluor488 (1:500) secondary antibodies in 0.3% PBS-Tx for 2h at RT for dSPN or iSPN classification. CTIP2 is expressed by SPNs and not interneurons (Arlotta et al., 2008) and PPE reliably labels iSPNs (Lee et al., 1997; Sharott et al., 2017). PPE antibody staining was facilitated through antigen retrieval by heating sections at 80°C in 10 mM sodium citrate (pH 6.0) for approximately 30-60 min prior to incubation with the PPE primary antibody. Occasionally the endogenous fluorescence would be boosted with antibodies against GFP (1:1000, chicken) or TdTomato (1:1000; rat) or slices were co-stained with the nuclear marker 4′,6-diamidino-2-phenylindole (DAPI) in PBS (1:100,000) to facilitate the delineation of brain structures. After classification of SPNs, the slices were washed 3 times in PBS and processed for DAB immunohistochemistry using standard procedures. Whole-brain fixation of embryonic and adult IUE brains was performed by rapid decapitation of the head and submersion in oxygenated sucrose cutting solution before submersion in 4% paraformaldehyde in 0.1 M phosphate buffer (PB; pH 7.4). The brains were fixed for 24 – 48 hours, after which they were washed in PBS. Whole-brain tissue was either directly (for postnatal tissue), or after embedding in 5% agar (for embryonic tissue), sectioned at 50 μm on a vibrating microtome (VT1000S; Leica Microsystems). All sections were pre-incubated in 10%–20% normal donkey serum (NDS; Jackson ImmunoResearch) or normal goat serum (NGS; Vector Laboratories) in PBS for more than 1h at RT. GFP^+ ($T\alpha1^+$) and $TdTom^+$ ($T\alpha1^-$) progenitors and neurons were predominantly visualized without antibody-mediated augmentation of fluorescence, but in rare cases the endogenous fluorescence was boosted with antibodies against GFP (1:1000, chicken) or TdTomato (1:1000; rat) and goat-anti-chicken AlexaFluor488 (1:500; Life Technologies) and goat-anti-rat AlexaFluor555 (1:500; Life Technologies).

Embryonic tissue was co-stained in 1:100,000 DAPI in PBS to facilitate the delineation of brain structures and/or labeled for the mitotic marker phosphohistone H3 (pH3) expressed by neural progenitors (1:500; rabbit). Adult tissue was co-stained in 1:100,000 4',6-Diamidino-2-Phenylindole (DAPI) in PBS to facilitate the delineation of brain structures and/or labeled for the μ-opioid receptor (MOR, 1:3000, goat), COUP TF1-interacting protein 2 (CTIP2, 1:1000, rat), dopamine- and cAMP-regulated phosphoprotein (DARPP-32, 1:250, mouse), parvalbumin (PV, 1:1000 guinea-pig), neuropeptide Y (NPY, 1:500, rabbit), calretinin (CR, 1:1000, rabbit) and/or pre-proenkephalin (PPE, 1:1000, rabbit) with corresponding secondaries (all 1:500). DARPP-32, NPY and PPE staining was facilitated through antigen retrieval by heating as described above. All sections were mounted in Vectashield (Vector Laboratories).

Stereology and analysis of tissue

Fluorescence images were captured with an LSM 710 confocal microscope using ZEN software (Zeiss) or Leica DM5000B epifluorescence microscope using Openlab software (PerkinElmer). Counting of labeled GFP^+ and $TdTom^+$ progenitors and young neurons and assessing their location within the embryonic brain was performed using ImageJ on z stacks of ~40 μm thickness. The reported counts per embryonic brain were obtained by averaging counts from 2-3 sections to increase accuracy. In embryonic tissue yellow cells could be seen occasionally, which were counted as GFP^+ and were assumed to have undergone recombination relatively recently. Positive cells had a fluorescence signal that was at least twice the background fluorescence (measured from randomly selected regions of the tissue). x- and y-coordinates of labeled cells were used to calculate both the distance from the ventricle and spread. Counting of progenitor cell basal processes was performed in z stack projections of confocal stacks of ~40 μm thickness. All clearly delineated processes above the subventricular zone and extending toward the pial surface were counted. M-phase reentry after IUE for aIP and OP was estimated through labeling of dividing progenitors with pH3 in tissue fixed at varying time-delays after IUE (Stancik et al., 2010). Localizing GFP^+ and $TdTom^+$ progenitors and young neurons in various sub-regions of the LGE was performed using a combination of anatomical landmarks (Schambra and Schambra, 2008) and previous delineations (Flames et al., 2007). Olfactory bulb analysis was performed using a total of 5 brains and all GFP^+ and $TdTom^+$ cells were counted in z stacks of ~40 μm thickness. Progenitor-derived SPN counting and co-localization analysis for CTIP2, DARPP-32, PPE, PV, CR and NPY was performed similar to Garas et al. (2016, 2018) and reported counts per brain were obtained by averaging counts from 2-3 sections to increase accuracy. In brief, a version of design-based stereology, the 'modified optical fractionator', was used to generate unbiased cell counts and map distributions of neurons in rostral, middle and caudal sections of striatum (Franklin and Paxinos, 2008). Once the chosen striatal coronal planes were identified and the immunofluorescence protocols carried out, the dorsal striatum was delineated using a Zeiss Imager M2 epifluorescence microscope (Carl Zeiss) equipped with a 20X (Numerical Aperture = 0.8) objective and StereoInvestigator software (MBF Biosciences). Imaging was subsequently performed by capturing a series of completely tessellated z stacked images (each 1 μm thick) at depths from 2 to 12 μm from the upper surface of each section at the level of the striatum (thereby defining a 10 μm-thick optical dissector). As counts were performed across the entirety of the striatum within a given rostro-caudal plane, the grid size and counting frame were set to the same size of 420 × 320 mm. To minimize confounds arising from surface irregularities, neuropil within a 2 μm 'guard zone' at the upper surface was not imaged. A neuron was counted if the top of its nucleus came into focus within the dissector. If the nucleus was already in focus at the top of the 10 μm-thick optical dissector the neuron was excluded. Normalized positions were calculated as described in Garas et al. (2016), 2018. Mediolateral and dorsoventral bias within each individual section was assessed by computing a Wilcoxon Sign rank test on the positions of all neurons across or within groups to test whether they significantly differed from zero (minimum 8 neurons for a given section). In Figure 1K a dot represents the normalized average position of all labeled SPNs in a single section. Mediolateral and dorsoventral positions of GFP^+ and $TdTom^+$ neurons across animals were compared by computing a Wilcoxon sign rank test on the normalized position in each direction for each section, when there was a minimum of 8 neurons of each type in a single section. In Figure 1K a line connects the average position of labeled SPNs from the same section. DAB-immunoreactive SPNs were visualized on a brightfield microscope and were reconstructed and analyzed using Neurolucida and Neuroexplorer software (MBF Biosciences). Only labeled SPNs that exhibited a full dendritic arbor were included for analysis e.g., cells with clear truncations were not included in the dataset. Scholl analysis and polarity analysis were performed using standard procedures. In brief, both Scholl and polarity plots were generated for individual SPNs by calculating the total dendritic length located within 10° segments with increasing distance from the soma. The dendritic lengths were subsequently normalized for an individual SPN and averaging the normalized plots of individual neurons generated final plots. Spines were manually counted on at least two randomly selected dendritic segments from individual neurons per dendritic class (e.g., secondary dendrite) and averaged. SPN reconstructions have been deposited on https://Neuromorpho.org.

QUANTIFICATION AND STATISTICAL ANALYSIS

Statistical details of experiments can be found in the respective Results sections and figure legends. All data are presented as means ± SEM. The 'n' refers to the number of animals/brains and 'n/n' refers first to the number of neurons and second to the number of animals/brains used. Statistical tests were all two-tailed and performed using SPSS 17.0 (IBM SPSS statistics) or GraphPad Prism version 5.0 (GraphPad software). Synaptic incidence ratios were compared with Fisher's exact test. Continuous data were assessed for normality using Kolmogorov-Smirnov and Shapiro-Wilk tests and appropriate parametric (ANOVA, paired t test and unpaired t test) or non-parametric (Mann-Whitney U, Wilcoxon Sign Rank and Kruskal-Wallis) statistical tests were applied (* $p < 0.05$, ** $p < 0.01$, *** $p < 0.001$).

Cell Reports, Volume *35*

Supplemental information

Diversity in striatal synaptic circuits arises from distinct embryonic progenitor pools in the ventral telencephalon

Fran van Heusden, Anežka Macey-Dare, Jack Gordon, Rohan Krajeski, Andrew Sharott, and Tommas Ellender

Supplemental Figures

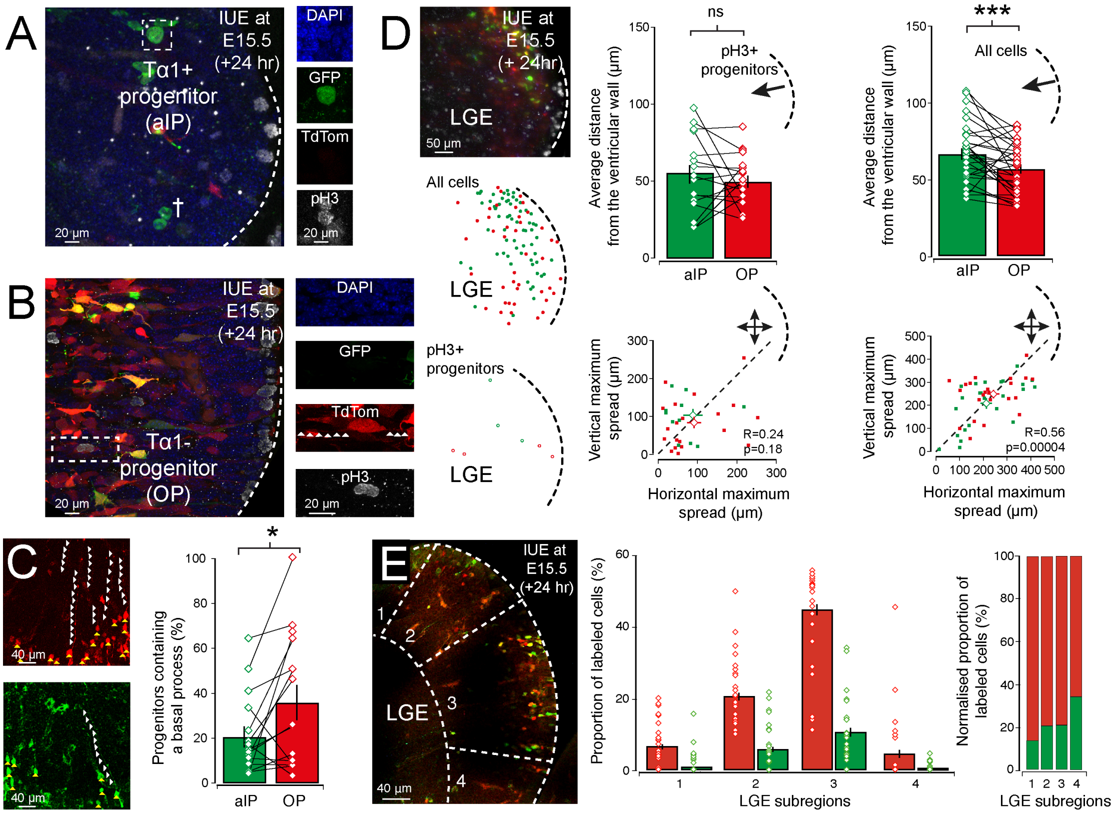

Figure S1: Spatial distribution and morphology of IUE labeled cells in the LGE. Related to Figure 1. (**A**) Actively dividing Tα1^{+}/GFP^{+} progenitors with a short rounded morphology and lacking a basal process, referred here as apical intermediate progenitors (aIP), could be found at both the ventricular wall or, as shown here, also at a slight distance from the ventricular wall, reminiscent of subapical progenitors (Pilz *et al.*, 2013). Cross symbol indicates Tα1^{+}/GFP^{+} progenitor undergoing cytokinesis. (**B**) Dividing Tα1^{-}/TdTom^{+} progenitors, referred here as other progenitors (OP), often retained a basal process during division and could similarly be found at the ventricular wall or, as shown here, at a distance from the ventricular wall, reminiscent of bipolar radial glial cells (Pilz *et al.*, 2013). (**C**) Quantification of progenitors in the LGE proliferative zones exhibiting a basal process. Note that significantly more Tα1^{-}/TdTom^{+} cells retained a basal process. (**D**) Measurements of the average distance from the ventricular wall and overall spread for Tα1^{+}/GFP^{+} and Tα1^{-}/TdTom^{+} cells within the LGE. We find that both Tα1^{+}/GFP^{+} and Tα1^{-}/TdTom^{+} cells are intermixed in the LGE (left). These cells consist of both dividing progenitors expressing the mitotic marker phospho-histone H3 (pH3^{+}) and non-dividing progenitors and young neurons (pH3^{-}). Whereas there is no difference in the average distance from the ventricular wall amongst dividing (pH3^{+}) Tα1^{+}/GFP^{+} and Tα1^{-}/TdTom^{+} progenitors (middle), the Tα1^{+}/GFP^{+} non-dividing progenitors and young neurons are located significantly further away from the ventricular wall 24h after IUE (right). (**E**) Example coronal section of embryonic LGE 24 hours after IUE at E15.5 with Tα1-Cre and FLEx plasmids demonstrating that GFP^{+} and TdTom^{+} labeled cells can be seen in all progenitor domains of the LGE. Bar plots of the average proportion of Tα1^{+}/GFP^{+} and Tα1^{-}/TdTom^{+} labeled cells in the different progenitor domains of the LGE (middle). Note that the labeling strategy predominantly targeted LGE3, but that all progenitor domains contain labeled cells (all labeled cells: LGE1: 7.6%, LGE2: 32.0%, LGE3: 59.0% and LGE4: 1.3%, n = 17 mice). The relative proportion of Tα1^{+}/GFP^{+} and Tα1^{-}/TdTom^{+} labeled cells appears relatively constant between the different domains of the LGE (right).

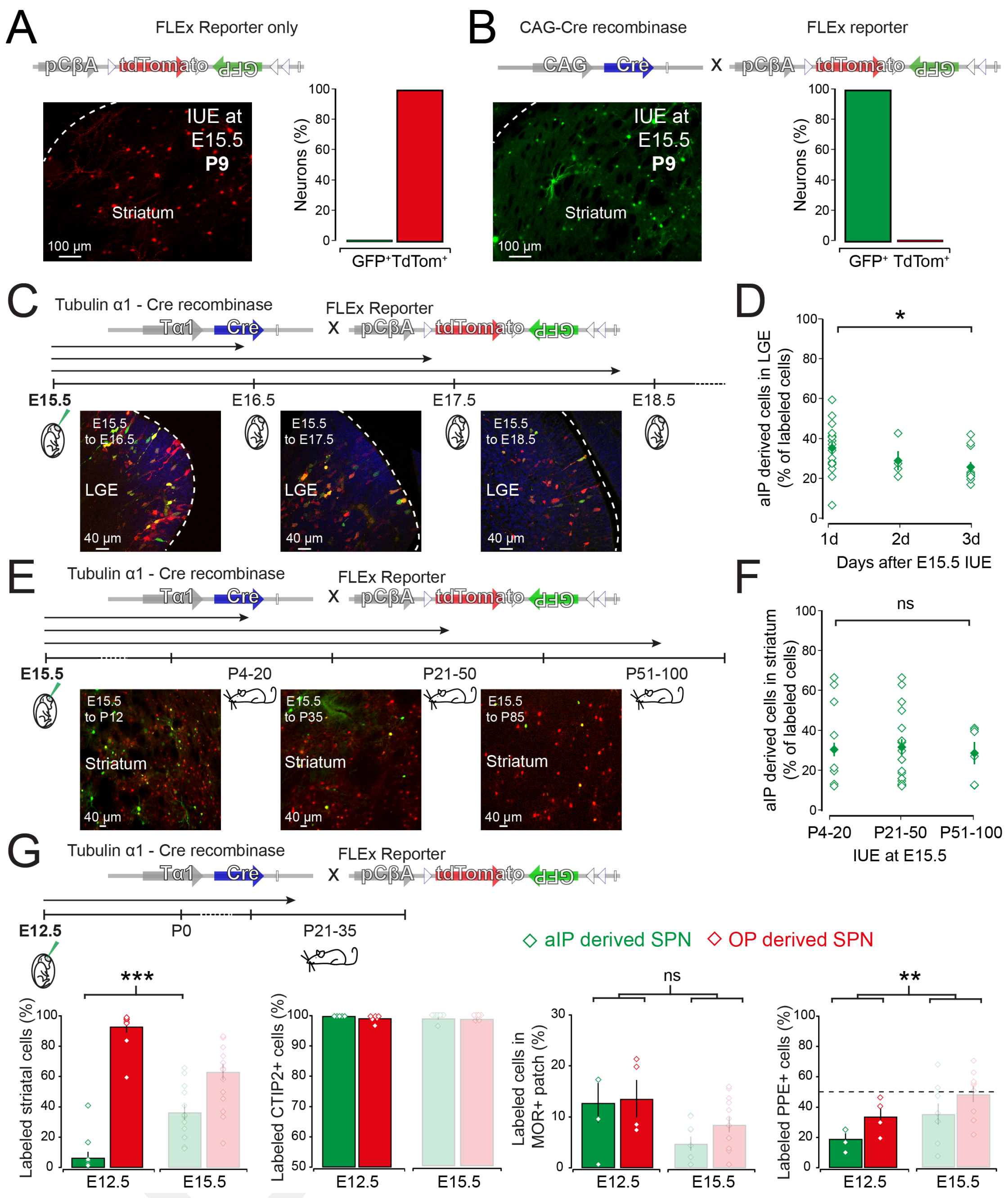

Figure S2: Faithful reporting of embryonic Tα1 expression. Related to Figure 1. (**A**) IUE at E15.5 of the CβA-FLEx reporter plasmid alone resulted in only TdTom$^+$ striatal SPNs at postnatal day (P)9 (n = 292/4 mice). This excludes the possibility of spontaneous recombination of the CβA-FLEx reporter. (**B**) IUE at E15.5 of the CβA-FLEx reporter plasmid and a second plasmid, in which a ubiquitous promoter ('CAG-Cre') drove Cre recombinase, resulted in complete and rapid recombination and only GFP$^+$ striatal SPNs were observed at P9 (n = 196/4 mice). (**C**) Tα1-Cre and CβA-FLEx plasmids were delivered by IUE at embryonic age E15.5 and GFP$^+$ and TdTom$^+$ cells were imaged in the LGE 24h, 48h or 72h later. (**D**) GFP$^+$ and TdTom$^+$ cells were quantified in LGE at 1d, 2d or 3d after IUE at E15.5. In support of the idea that the majority of Cre-mediated recombination of the reporter plasmid occurred within 24 h following IUE, the proportion of GFP$^+$ cells in the LGE remained relatively stable during embryonic development with a small but significant decrease in the number of GFP$^+$ cells (p = 0.045, Kruskal-Wallis test; n = 1204/17, 535/4 and 620/11 at 1d, 2d and 3d after IUE at E15.5). (**E**) GFP$^+$ and TdTom$^+$ striatal neurons were imaged across a range of postnatal ages after IUE at E15.5. (**F**) The proportion of GFP$^+$ aIP-derived striatal neurons did not change significantly across a wide range of postnatal ages (p = 0.99, Kruskal-Wallis test, n = 3743/20, 2677/24 and 345/6 mice at

P4-20, P21-50, P51-100) after IUE at E15.5, consistent with the idea that there was no further recombination of the reporter plasmid postnatally. (**G**) IUE at the early embryonic age E12.5 of Tα1-Cre and CβA-FLEx plasmids resulted in significantly lower numbers of GFP^+ aIP-derived striatal neurons in postnatal (P21-35) striatum (E12.5: 6.6 ± 4.0% and E15.5: 31.6 ± 3.4%, Mann-Whitney test, p=0.0002, n = 657/10 and 2677/24 mice) as compared to IUE at E15.5, supporting the idea that Tα1-expressing progenitors form a considerable proliferating population during later periods of neurogenesis. The majority of progenitor derived neurons labeled at this embryonic age expressed the SPN marker CTIP2 (E12.5 aIP: 100.0 ± 0.0% and E12.5 OP: 99.2 ± 0.5%, n = 275/4 mice) and could be found in both (MOR)-rich patches and MOR-poor matrix compartments (E12.5 aIP/MOR^+: 12.8 ± 3.9% and OP/MOR^+: 13.6 ± 3.7%, 370/4 mice, middle) with a trend towards larger numbers of early labeled SPNs in (MOR)-rich patches (E12.5: 12.4 ± 2.4% vs. E15.5: 6.9 ± 1.3% in MOR^+ patches; Mann-Whitney test, p = 0.170, n = 370/4 and 427/12 mice). Grouped together the E12.5 IUE labeled SPNs contained lower numbers of PPE^+/iSPNs as compared to those labeled at E15.5 (E12.5 vs. E15.5, Mann-Whitney test; p = 0.006; n = 275/4 and 254/9 mice, right) consistent with the idea that dSPNs have an earlier birthdate.

DRAFT

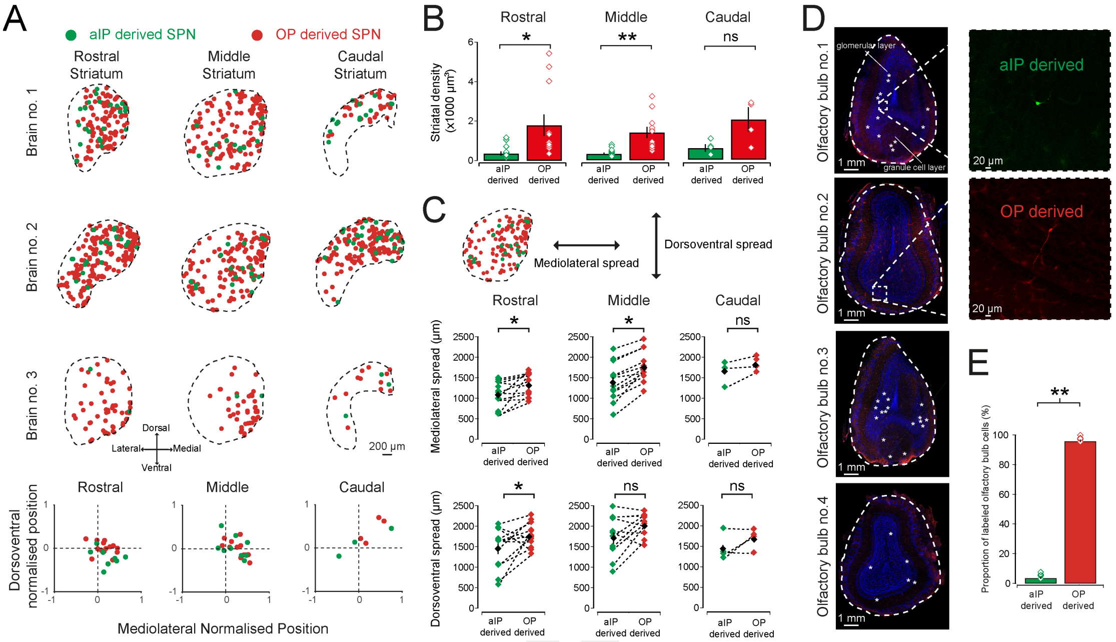

Figure S3: Labeled aIP and OP-derived striatal SPNs in the rostral, central, and caudal striatal regions and olfactory bulb. Related to Figure 1. (**A**) Distribution of aIP and OP-derived SPNs (P21-35) in the rostral, middle, and caudal regions of the striatum in three different IUE brains. Labeled progenitor-derived SPNs were predominantly found in the medial aspects of the striatum. Labeled SPNs were only counted within a 10-µm plane of a brain section (see **Methods**), which for the dataset reported in this figures was on average 13.8 ± 1.9 aIP-derived SPNs and 60.5 ± 9.4 OP-derived SPNs per 10-µm plane of a brain section. (**B**) The average relative densities of aIP and OP-derived SPNs in rostral, middle, and caudal regions of striatum were similar, with an overall higher density of OP-derived SPNs. (**C**) Analysis of the maximum spread of aIP and OP-derived SPNs in the mediolateral and dorsoventral axes shows that OP-derived SPNs exhibit a larger spread than aIP-derived SPNs in both axes. (**D**) IUE of embryonic progenitors in the LGE also labeled a small number of, predominantly OP-derived, olfactory bulb cells. Example images of four different olfactory bulbs from IUE mice with labeled cells indicated by asterisks. High magnification images of an aIP-derived cell (right, top) and OP-derived cell (right, bottom) in the olfactory bulb. (**E**) The majority of labeled cells in the olfactory bulbs were OP-derived (aIP: 3.9 ± 1.6% and OP: 96.1 ± 1.6%, p=0.0079, Mann-Whitney test, n= 191/ 5 olfactory bulbs).

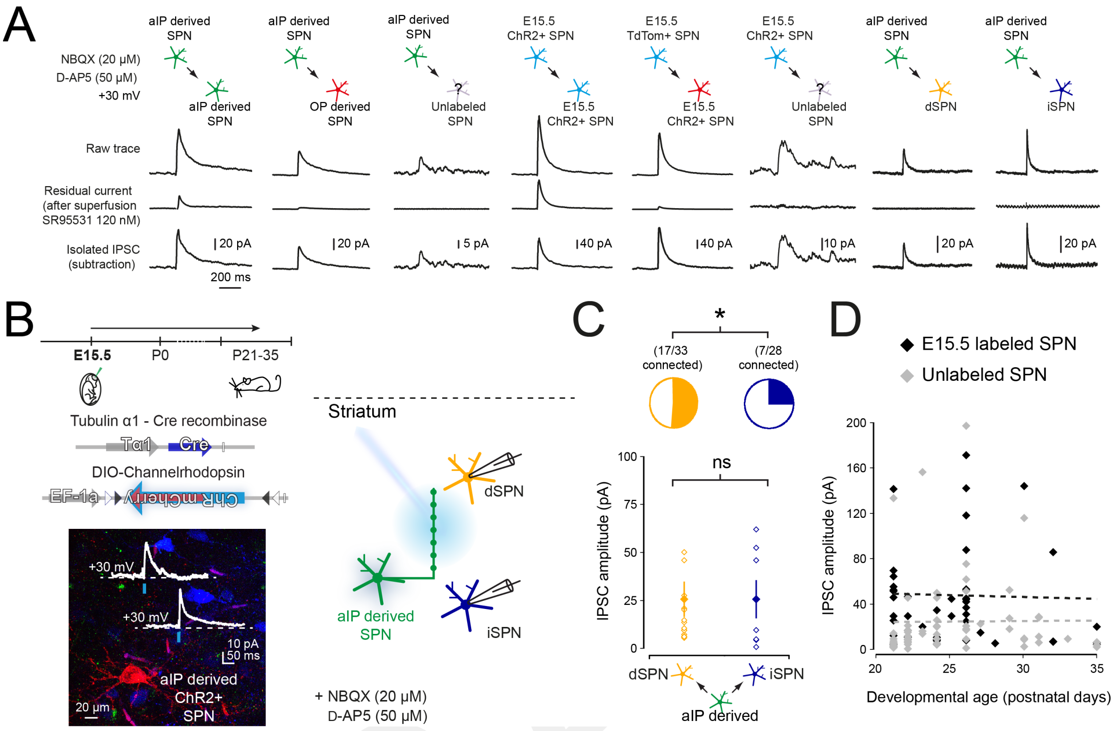

Figure S4: Optogenetic study of local inhibitory connections between SPNs. Related to Figure 4. (**A**) Example responses recorded from striatal SPNs after photo-activation of ChR2-expressing presynaptic SPNs. All recordings were made at a holding potential of +30 mV in the presence of blockers of excitatory transmission. Plotted are the raw responses to photo-activation (top), the residual responses to photo-activation after additional superfusion of low concentrations of the $GABA_A$-receptor antagonist SR95531 (200nM) (middle), and the subtracted responses which corresponded to the $GABA_A$ receptor-mediated response or IPSC used for comparisons of inhibitory synaptic strength (bottom). (**B**) IUE of Tα1-Cre and DIO-ChR2-mCherry plasmids allowed for the expression of ChR2-mCherry in aIP-derived neurons (P21-35). Diagram of the experimental setup consisting of whole-cell voltage-clamp recordings of dSPNs and iSPNs while stimulating aIP-derived neurons with a 473 nm wide-field LED (right). Immunohistochemical staining (bottom, left) of two recorded and labeled SPNs as revealed with streptavidin-405 (in blue) in proximity to an aIP-derived ChR2-mCherry expressing SPN (in red). Inset: example current traces of evoked pharmacologically isolated IPSCs in response to brief light pulses. (**C**) aIP-derived striatal neurons exhibited a slight but significantly higher incidence of connecting to dSPNs (53%), but optical activation of aIP-derived neurons resulted in similar amplitude postsynaptic IPSCs in both dSPNs and iSPNs. (**D**) Plot of IPSC amplitude across developmental age for both E15.5 progenitor derived SPNs (black) and unlabeled SPNs (grey; unknown birthdate or progenitor origin). Dashed line is a linear fit to data suggesting that the increased strength of connections between SPNs of matched neurogenic stages is retained across the postnatal developmental time investigated.

Supplemental Tables

	aIP derived	OP derived	p-value	Unlabeled	ChR2+ aIP derived
Resting membrane potential (mV)	-78.47 ± 1.06	-80.20 ± 0.85	0.232	-80.58 ± 0.94	-79.61 ± 1.77
Input resistance (MΩ)	103.57 ± 3.92	97.22 ± 3.62	0.154	95.42 ± 5.36	101.43 ± 8.32
Membrane time constant (ms)	3.41 ± 0.22	2.72 ± 0.15	**0.009**	2.64 ± 0.17	3.45 ± 0.48
Spike threshold (mV)	-39.64 ± 0.82	-37.11 ± 0.83	**0.039**	-36.92 ± 1.24	-38.23 ± 0.92
Spike rate (500pA) (Hz)	38.61 ± 3.33	38.92 ± 3.61	0.953	36.53 ± 2.79	35.19 ± 6.97
Spike rate (400pA) (Hz)	35.71 ± 2.61	36.65 ± 3.01	0.824	34.22 ± 2.21	32.79 ± 4.45
Spike rate (300pA) (Hz)	31.68 ± 2.10	32.03 ± 2.51	0.919	29.00 ± 2.32	31.83 ± 2.65
Spike rate (200pA) (Hz)	23.40 ± 2.24	20.44 ± 2.10	0.348	22.39 ± 1.98	22.60 ± 3.02
Spike rate (100pA) (Hz)	12.81 ± 1.55	15.45 ± 3.40	0.440	6.56 ± 1.24	8.50 ± 1.70
First ISI (ms)	19.14 ± 1.57	20.57 ± 1.41	0.506	21.62 ± 1.79	19.85 ± 2.37
Second ISI (ms)	20.75 ± 1.38	22.00 ± 1.54	0.565	21.93 ± 1.35	19.44 ± 2.01
Third ISI (ms)	26.37 ± 1.79	26.62 ± 2.07	0.932	26.63 ± 1.73	26.32 ± 3.40
Fourth ISI (ms)	28.90 ± 1.60	27.57 ± 1.64	0.576	28.52 ± 1.70	25.82 ± 2.54
First spike amplitude (mV)	80.40 ± 1.61	77.07 ± 1.88	0.207	84.45 ± 2.47	75.03 ± 2.92
Second spike amplitude (mV)	64.34 ± 2.28	64.52 ± 2.16	0.954	72.45 ± 2.41	59.04 ± 3.41
First spike duration (ms)	1.83 ± 0.09	1.91 ± 0.13	0.633	1.74 ± 0.07	1.70 ± 0.15
Second spike duration (ms)	2.62 ± 0.16	2.53 ± 0.12	0.639	2.38 ± 0.11	2.63 ± 0.29

Data are given as mean ± SEM, statistical comparsions by *t*-test or Mann-Whitney U test

DRAFT

Table S1: Intrinsic membrane properties of striatal SPNs. Related to Figures 2 and 4.

	aIP derived	OP derived	p-value	Unlabeled
Soma surface area (µm^2)	482.27 ± 24.35	629.20 ± 51.60	**0.02**	671.47 ± 134.45
Number of Dendrites	6.82 ± 0.62	6.07 ± 0.33	0.08	5.38 ± 0.31
Total Dendritic Length (µm)	2177.73 ± 232.39	2346.70 ± 148.97	0.53	2162.70 ± 120.75
Average Dendrite Length (µm)	324.76 ± 24.92	398.20 ± 29.41	**0.03**	421.24 ± 26.54
Number of Branch Points	19.55 ± 1.65	16.20 ± 1.40	**0.05**	15.35 ± 0.88

Data are given as mean ± SEM, statistical comparsions by *t*-test or Mann-Whitney U test

Table S2: Morphological properties of striatal SPNs. Related to Figure 2.

DRAFT

Medial prefrontal cortex	aIP derived	OP derived	p-value	Unlabeled
Amplitude (mV)	3.77 ± 1.00	1.46 ± 0.59	4.75E-2	2.80 ± 0.60
Duration (ms)	161.83 ± 31.50	143.56 ± 44.30	0.46	125.92 ± 18.81
Rise time (ms)	6.64 ± 0.75	4.35 ± 1.01	0.68	6.46 ± 0.85
Decay time (ms)	75.28 ± 14.74	68.68 ± 22.09	0.33	57.08 ± 8.82
**Visual cortex**				
Amplitude (mV)	0.67 ± 0.27	3.54 ± 0.96	1.03E-4	2.80 ± 0.54
Duration (ms)	135.40 ± 21.15	155.53 ± 13.32	0.48	167.14 ± 19.85
Rise time (ms)	6.29 ± 1.08	6.22 ± 0.72	0.72	8.14 ± 0.62
Decay time (ms)	59.90 ± 10.33	72.55 ± 5.86	0.23	73.43 ± 9.88

Data are given as mean ± SEM, statistical comparisons by *t*-test.

Table S3: Synaptic EPSP properties to ChR2-mediated activation of cortical afferents (combined paired and unpaired recordings). Related to Figure 3.

DRAFT

	aIP derived to aIP derived	aIP derived to OP derived	p-value	aIP derived to dSPN	aIP derived to iSPN	p-value	aIP derived to Unlabeled
Amplitude (pA)	55.05 ± 15.29	48.70 ± 16.95	0.74	25.48 ± 9.28	25.55 ± 10.03	0.71	31.37 ± 12.14
Duration (ms)	137.50 ± 20.46	157.69 ± 25.18	0.50	111.81 ± 11.78	97.44 ± 15.87	0.46	117.46 ± 21.86
Rise time (ms)	6.85 ± 1.16	6.76 ± 1.03	0.77	4.37 ± 0.67	5.06 ± 1.41	0.90	6.79 ± 1.68
Decay time (ms)	64.28 ± 9.70	77.81 ± 11.42	0.25	57.06 ± 7.91	46.06 ± 9.43	0.38	53.74 ± 10.19

Data are given as mean ± SEM, statistical comparisons by Mann-Whitney U test

Table S4: Synaptic IPSC properties from aIP derived SPNs. Related to Figure 4 and Supplemental Figure 4.
